# WDR5 inhibition halts metastasis dissemination by repressing the mesenchymal phenotype of breast cancer cells

**DOI:** 10.1186/s13058-019-1216-y

**Published:** 2019-11-21

**Authors:** Simona Punzi, Chiara Balestrieri, Carolina D’Alesio, Daniela Bossi, Gaetano Ivan Dellino, Elena Gatti, Giancarlo Pruneri, Carmen Criscitiello, Giulia Lovati, Marine Meliksetyan, Alessandro Carugo, Giuseppe Curigliano, Gioacchino Natoli, Pier Giuseppe Pelicci, Luisa Lanfrancone

**Affiliations:** 1 Department of Experimental Oncology, European Institute of Oncology IRCCS, Milan, Italy; 2 Humanitas University, Pieve Emanuele (MI), 20090 Italy; 3 Humanitas Clinical and Research Institute, Rozzano (MI), 20089 Italy; 4 Present address: Department of Internal Medicine and Medical Specialties (Di.M.I), University of Genova, Genoa, Italy; 5 Present address: Institute of Oncology Research (IOR), Bellinzona, Switzerland; 6 Department of Oncology and Hemato-Oncology, University of Milan, Milan, Italy; 7 Department of Pathology, Biobank for Translational Medicine Unit, European Institute of Oncology, IRCCS, Milan, Italy; 8 Present address: Istituto Nazionale dei Tumori – Fondazione IRCCS, Milan, Italy; 9 Division of Early Drug Development for Innovative Therapy, European Institute of Oncology IRCCS, Milan, Italy; 10 Institute for Applied Cancer Science, UT MD Anderson Cancer Cente, Houston, TX 77030 USA

**Keywords:** Breast cancer, EMT, Metastasis, TGFβ1, WDR5

## Abstract

**Background:**

Development of metastases and drug resistance are still a challenge for a successful systemic treatment in breast cancer (BC) patients. One of the mechanisms that confer metastatic properties to the cell relies in the epithelial-to-mesenchymal transition (EMT). Moreover, both EMT and metastasis are partly modulated through epigenetic mechanisms, by repression or induction of specific related genes.

**Methods:**

We applied shRNAs and drug targeting approaches in BC cell lines and metastatic patient-derived xenograft (PDX) models to inhibit WDR5, the core subunit of histone H3 K4 methyltransferase complexes, and evaluate its role in metastasis regulation.

**Result:**

We report that WDR5 is crucial in regulating tumorigenesis and metastasis spreading during BC progression. In particular, WDR5 loss reduces the metastatic properties of the cells by reverting the mesenchymal phenotype of triple negative- and luminal B-derived cells, thus inducing an epithelial trait. We also suggest that this regulation is mediated by TGFβ1, implying a prominent role of WDR5 in driving EMT through TGFβ1 activation. Moreover, such EMT reversion can be induced by drug targeting of WDR5 as well, leading to BC cell sensitization to chemotherapy and enhancement of paclitaxel-dependent effects.

**Conclusions:**

We suggest that WDR5 inhibition could be a promising pharmacologic approach to reduce cell migration, revert EMT, and block metastasis formation in BC, thus overcoming resistance to standard treatments.

## Background

Despite recent advances in breast cancer (BC) treatment, women frequently develop resistance to endocrine and chemotherapies and die of metastasis [1] so that, for these patients, new treatment strategies are mandatory. The reversion of epithelial-to-mesenchymal transition (EMT) through re-differentiation of cancer cells represents a potential therapeutic challenge to ameliorate patients’ prognosis [2, 3]. The EMT is a well-defined developmental program adopted by tumor cells during the metastatic cascade to gain migratory ability and reach distant organs, losing epithelial cell adhesion and cell-cell contacts, while undergoing cell shape remodeling and cytoskeleton rearrangement [4]. Concurrently, the expression of epithelial markers is inhibited, in favor of an increase in the expression of the mesenchymal genes [5]. The epithelial and mesenchymal states represent two opposite cellular phenotypes that the cells reach going through several intermediary phases [6]. During EMT, changes in gene expression are crucial for this process to occur.

The transforming growth factor β1 (TGFβ1) signaling pathway is involved in a plethora of events regulating EMT. TGFβ1 is secreted by tumor cells as well as by cells of the surrounding stroma, and is crucial in the regulation of distinct processes, as cytoskeleton organization, survival, cell migration, and invasion [7]. TGFβ1 signaling may control EMT and metastasis by sustaining the epigenetic machinery through the DNA binding activity of DNMT1 [8] or the histone methylation-coupled transcriptional activation or repression of PRMT5-MEP50 axis [9]. Furthermore, numerous epigenetic modifiers (i.e., HDACs, LSD1, SET8, PRC1/2, PRMT7, and BRG1) seem to give a great contribution to such a modulation since histone modifications (acetylation/deacetylation and methylation/demethylation) are implicated in either inducing or repressing specific sets of EMT-related genes (SNAI1/2, TWIST1/2 and ZEB1/2) [10, 11].

WDR5 is a WD40 repeat protein that recognizes the histone H3 amino-terminal tail and is essential for lysine 4 (H3K4) methylation [12]. A large body of evidence supports the pivotal role of WDR5 in tumor growth and proliferation [13–18], differentiation [19, 20], and metastasis [21–23], and suggests that its expression is prognostic in different tumor types [13, 15, 24–27]. Moreover, WDR5 binds the mesenchymal gene promoter and transcriptionally regulates N-cadherin in BC upon hypoxia treatment [28], ZNF407 in colorectal cancer [23], HOXA9 in prostate cancer [22], and SNAIL1 and VIMENTIN in lung tumor cells [9], leading to EMT. Despite its proven involvement in EMT, a direct interplay of WDR5 and TGFβ1 in activating this process in BC remains elusive.

Here, we demonstrate that WDR5 is involved in EMT and metastasis in BC and that its inhibition, by reducing the migratory and mesenchymal phenotype, drives the cells toward an epithelial-like status. Our data indicate a direct regulation of WDR5 on the TGFβ1 pathway. Moreover, we suggest that WDR5 inhibition could be used as a therapeutic approach in Triple Negative (TN) and Luminal B (LB) metastatic breast cancer and that its combination with chemotherapy may be advantageous for treatment of chemo-resistant patients.

### Materials and methods

#### PDX tissue bank generation

Patients enrolled in the study were selected on the basis of highly aggressive metastatic disease diagnoses (Luminal B and Triple Negative subtypes) and resistance to different lines of therapy. Biopsies from liver and lung were transplanted in Matrigel (Corning #356231) orthotopically in the fourth mammary gland of female NSG mice, as previously described [29]. Tumors were monitored weekly and serially passaged in NSG after tissue digestion (see “PDX culture”). Tumors were characterized by IHC on the basis of the prognostic clinical markers, i.e., estrogen, progesterone, HER2, and Ki67, by pathologists and compared to patient tumors. Positive staining is expressed as percentage.

### Animals

Non-obese diabetic/severe combined immunodeficiency (NOD/SCID) mice were purchased from Harlan Laboratories. NOD.Cg-Prkdc^scid^ Il2rg^tm1Wjl^/SzJ (NSG) mice were purchased from Charles River. Only female mice 6–12 weeks old (15–20 g weight) were used for experimental procedures.

### Ethic statements

Investigations have been conducted in accordance with the ethical standards and according to National and International guidelines. In vivo studies were performed after approval from our fully authorized animal facility, notification of the experiments to the Ministry of Health (as required by the Italian Law) (IACUCs N° 757/2015, 1246/2015 and 327/2016) and in accordance to EU directive 2010/63. Human tissue biopsies were collected from patients whose informed consent was obtained in writing according to the policies of the Ethics Committee of the European Institute of Oncology and regulations of Italian Ministry of Health. The studies were conducted in full compliance with the Declaration of Helsinki.

### PDX culture

PDX tumors were dissociated by enzymatic and mechanical digestion (Miltenyi Biotec), and cells were plated to obtain short-term cultures. PDX cells were maintained in DMEM/F12 (1:1, Lonza/Gibco) supplemented with 10% fetal bovine serum (FBS) (HyClone, GE Healthcare Life Science), 10 mM HEPES (Sigma), 5 μg/mL insulin (Roche), 0.5 μg/mL hydrocortisone (Sigma), 10 ng/mL epidermal growth factor (EGF; Tebu-Bio), and 50 ng/mL Cholera Toxin (Sigma).

### Cell lines

Experiments were performed in MCF10DCIS.com (from Wayne State University, 5057 Woodward Avenue, Detroit, Michigan), SUM149PT (from Asterand), HCC1428 (from ATCC), MDAMB468 (from CLS), ZR751 (from ATCC), and MDA-MB-231 and MCF10A (both from NIH Institute). Cell lines were maintained in their respective media as recommended by suppliers. Cell line authentication was performed in house by Gene Print 10 System every 6 months (Promega). All cell lines were tested for mycoplasma and resulted negative.

### In vivo study

PDXs, MCF10DCIS.com, SUM149PT, HCC1428, MDAMB468, and ZR751 were infected with control shRNA (shLuc) and two pooled shWDR5. 2 × 10^5^ infected cells were orthotopically injected into the fourth mammary gland of three to nine mice (PDXs cells, SUM149PT, HCC1428, MDAMB468, ZR751 in NSG mice; MCF10DCIS.com in NOD/SCID), according to the experimental setting. Tumor volume was calculated using this formula: V = L × l^2^/2 (L length; l width). MDA-MB-231 cells were double transfected to express luciferase (Addgene 17477) and to silence a neutral control (SCR) or WDR5. Then 2 × 10^5^ cells were transplanted in the fourth mammary gland of 12 NSG mice *per* group. The mice were monitored for primary tumor growth. For metastasis experiments, when a volume of about 0.5 cm^3^ was reached, tumors were excised and mice monitored weekly for metastasis formation. Luciferase expression was assessed by bioluminescence imaging (IVIS Lumina Imaging System - PerkinElmer) and mice were sacrificed when lungs or axillary lymph nodes resulted positive to luminescence. Luminescence was quantified by using Living Image software and expressed as radiance in photons of the region of interest.

### In vitro study

Proliferation, FBS-directed migration on Boyden chamber, wound healing, and time-lapse live cell random migration assays were performed as described in Additional file 3: Supplementary Methods.

### Immunofluorescence

MCF10DCIS.com or MDA-MB-231 cells, infected to silence WDR5 or treated by drugs, were plated on slides and allowed to attach overnight. Next day, cells were fixed with 4% paraformaldehyde for 10 min, permeabilized with 0.01% Triton-X, and blocked for 1 h with 2% bovine serum albumin. The antibodies against the following protein were used: FITC-labeled Phalloidin (P5282), Vimentin [V9] (ab8069), CDH2 [5D5] (ab98952), CDH1 (24E10), SNAI2 (C19G7) and SNAI1 (C15D3), and α-Tubulin (T9026). Slides were then counterstained with 4′,6-diamidino-2-phenylindole (DAPI) for nuclei labelling and mounted on glass slides with Mowiol. Images were collected by motorized Olympus fluorescence microscope at × 40 magnification.

### Adhesion assay

For adhesion assay, 2 × 10^4^ shLuc and shWDR5 MCF10DCIS.com cells were plated onto different extracellular matrices (collagen—CL, laminin—LM, fibronectin—FN, matrigel—MG). After 1.5 h, cells were fixed and stained with 0.5% Crystal Violet. Three images *per* well were acquired, and cell number and area were quantified by using ImageJ software by manually delineating the edges of selected cells (a total of 30 measurements *per* group) and recording the circularity value.

### Western blot

PDX cells and other BC cell lines were lysed in RIPA buffer and processed, as previously described [30]. Membranes were probed with antibodies reported in Additional file 3: Supplementary Method. Images were cropped at specific protein band of interest to improve the clarity of data presentation.

### Survival and expression analysis

Association between WDR5 expression and metastasis-free survival (MFS) in 295 breast cancer patients was calculated using PROGgene V2 software on NKI publicly available data sets [31]. MFS were represented by Kaplan-Meier functions, and cohorts were divided at the median of gene expression. Statistic comparison between high and low expression groups was performed using log-rank test. Association between WDR5 silencing and survival in mice was calculated by using GraphPad Prism 5.0, and significative differences among groups were calculated by using log-rank test. Differences were considered significant at *P* < 0.05. Analysis on TCGA data set of breast cancer patients was performed by using publicly available data in cBioportal for Cancer Genomics by considering expression values of genes from RNA-seq. Overexpression was considered for *z*-score ≥ + 2. Co-occurrence of expression of each gene and WDR5 was represented by *P* values, calculated by the Fisher exact test.

### Drug treatment

MDA-MB-231 and MCF10DCIS.com cells were treated with a single exposure for 3 days of OICR-9429 (MD Anderson Cancer Center - Texas) at a final concentration of 1 μM–5 μM–10 μM or 20 μM for 3 days and then plated for migration assay. LY2157299 (galunisertib) (MCE, HY-13226) was added by a single administration for 3 days at final concentration of 10 μM. Short-term in vitro growth inhibition by drugs in PDXs and MDA-MB-231 cells was assessed by Cell Titer Glo (Promega). Briefly, PDX cells (obtained from third passage in mice) were thawed, plated in 2D cultures in 96 wells (5000 cells *per* well), and treated for 3 days by a single exposure to vehicle or concentrations of the following drugs: paclitaxel (1 nM–5 μM–10 nM), OICR-9429 20 μM, or galunisertib 10 μM alone or in combination. MDA-MB-231 cells (2000 cells *per* well) were treated for 3 days with paclitaxel (10 nM), OICR-9429 (20 μM), or galunisertib (10 μM) alone or in combination. The inhibition of viability is indicated as a percent over control cell viability of the aforementioned drugs (calculated using GraphPad Prism software).

### RNA sequencing

Total RNA was extracted from shLuc and shWDR5 MCF10DCIS.com or PDX cells by using the Maxwell 16LEV simply RNA tissue kit. mRNA purification and NGS libraries were obtained following Illumina instruction (TruSeq RNA Sample Preparation). Bioinformatic analysis is fully described in Additional file 3: Supplementary Methods.

### ChIP sequencing

ChIP lysates were generated from 10–15 × 10^6^ cells as reported previously [32]. ChIP DNA was prepared for HiSeq 2000 Illumina sequencing. Samples were aligned to human genome, and bioinformatic analysis is fully described in Additional file 3: Supplementary Methods.

### Data access

Data sets are available in the Gene Expression Omnibus (GEO) database under accession number GSE113289.

### Quantitative RT-PCR

Total RNA was extracted from PDXs, MCF10DCIS.com, and MDA-MB-231 and reverse transcribed using OneScript Plus Reverse Transcriptase and cDNA Synthesis kit (abm). Quantitative RT-PCR analyses were done on Biorad CFX Real-Time PCR System with the fast-SYBR Green PCR kit as instructed by the manufacturer (Applied Biosystems). The transcription level of the RPLP0 housekeeper gene was used as a normalizer. Complete primer sequences are reported in Additional file 2: Table S4.

### Statistical analysis

Data are represented as mean ± SD of biological triplicates (if not diversely indicated in the text). Comparisons between two or more groups were assessed by using two-tailed Student’s *t* test, one-way or two-way ANOVA followed by the Dunnett post test, or the Bonferroni post test, as indicated in figure legend. *P* < 0.05 and lower were considered significant. For RNA-seq and ChIP-seq analysis, statistical parameters are indicated in Additional file 3: Supplementary Methods.

## Results

### WDR5 promotes breast cancer growth in vivo

**Fig. 1:**
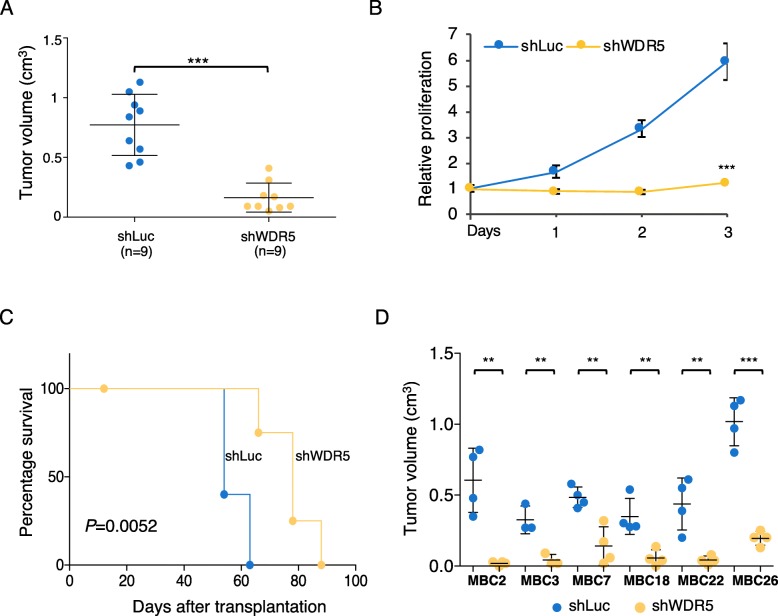
WDR5 controls tumorigenesis in breast cancer. **a** MCF10DCIS cell line was infected to silence WDR5 (shWDR5) or a neutral control (shLuc) and inoculated in vivo in immunocompromised mice. When tumors of control group reached maximum volume expected, all the mice were sacrificed to compare effect on tumor growth. Dot plots represent tumor volume in shWDR5 or shLuc MCF10DCIS (*n* = 9 *per* group; mean ± SD—cm^3^). Statistical significance was determined using an unpaired Student *t* test (***P < 0.001). **b** In vitro relative proliferation values of shWDR5 in MCF10DCIS cells are reported with respect to control (shLuc). Statistical significance on *n* = 3 experiments was determined using an unpaired Student *t* test (***P < 0.001). **c** Percentage of survival of mice belonging to control or shWDR5 groups was calculated. Mice were sacrificed when each tumor reached maximum volume expected. Differences among groups were calculated by using Log-rank test (*n* = 5 *per* group; *P* = 0.0052). **d** Six MBC PDXs were infected to target control (shLuc) and shWDR5. Transduced cells were transplanted in NSG mice. Dot plots represent volume (mean ± SD—cm^3^) of three to four tumors *per* group. Statistical significances were calculated by applying an unpaired Student *t* test (**P < 0.01; ***P < 0.001)

In our previous study, we performed a loss of function shRNA screening in the MCF10DCIS.com (hereafter MCF10DCIS) BC cell line to identify epigenetic targets driving tumorigenesis [30]. Since WDR5 was strongly depleted both in the in vivo and in vitro screens and it ranked as one of the best candidates in sustaining BC growth [30], we validated its oncogenic role in MCF10DCIS cells. First, we verified that two single shRNAs (sh1- or sh2-WDR5) in MCF10DCIS were able to specifically target and silence WDR5 (Additional file 1: Figure S1A). Then, MCF10DCIS cells were transduced with the two pooled WDR5 shRNAs and a corresponding control (shLuc) (Additional file 1: Figure S1A) and transplanted in NOD/SCID mice to assess in vivo tumor growth (*n* = 9 *per* group). WDR5 silencing strongly reduced tumor volume in vivo (*P* < 0.001; Fig. 1a), confirming its crucial role in sustaining BC growth. Accordingly, WDR5 silencing significantly reduced in vitro cell proliferation (Fig. 1b), as demonstrated in other tumor types [13, 15, 16, 18]. Analysis of survival rate among additional groups (*n* = 5 mice *per* group) revealed that WDR5 inhibition significantly increased disease-free survival (*P* = 0.0052), supporting the prognostic role of WDR5 in BC (Fig. 1c). Conversely, WDR5 overexpression increased tumor growth in MCF10DCIS cells (Additional file 1: Figure S1B-S1C), paralleling scattered evidences of correlation of WDR5 overexpression with tumorigenesis [13, 15, 24–27].

In order to reproduce human cancer in mouse, we created a cohort of patient-derived xenografts (PDXs) obtained from liver and lung metastases of BC (MBC) patients who developed resistance to different lines of therapy (Additional file 2: Table S1). We focused on LB and TN subtypes since they are the most aggressive, frequently metastasize to distant organs and few personalized approaches (with exception of hormone therapy) are available [33]. These PDXs recapitulated the phenotypic and molecular features of the tumors (Additional file 2 Table S2) and we used them as surrogate of patients with BC [29].

Six PDXs were used for the in vivo studies. Two TN (MBC2 and MBC7) and 4 LB (MBC3, MBC22, MBC18 and MBC26) PDXs were independently transduced with two shWDR5 in pool or a corresponding control (shLuc) (Additional file 1: Figure S1D). In parallel, we also used two TN (SUM149PT and MDAMB468) and two LB (HCC1428 and ZR751) cell lines (Additional file 1: Figure S1E). The reduction of WDR5 expression significantly inhibited tumor growth in MBC PDXs (Fig. 1d) and in TN and LB cell lines (Additional file 1: Figure S1F), suggesting that WDR5 is involved in tumorigenesis both in ER+ and ER− BC.

### WDR5 controls transcriptional changes in breast cancer

**Fig. 2:**
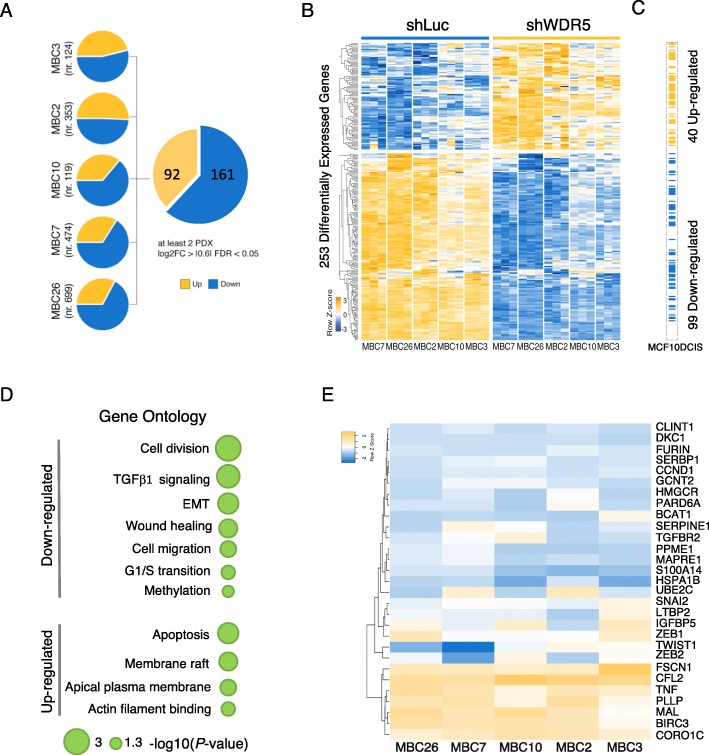
WDR5 regulates gene expression in breast cancer. RNA-seq was performed in 5 PDXs upon WDR5 silencing. **a** Pie charts showed differentially expressed genes (DEGs) obtained by pairwise analysis in each PDX comparing control (shLuc) and shWDR5 MBC PDXs cells. Genes were identified as DEGs when the following criteria were met: log_2_ fold change (FC) ≥ |0.6|, false discovery rate (FDR) < 0.05, and expression > 0.5 RPKM. DEGs in common at least in two PDXs were considered for further analysis. **b** Heatmap showed normalized expression values after removing batch effects of 253 DEGs identified as mentioned above (row scaled, *z*-score). **c** Corresponding genes which expression was significantly modified in MCF10DCIS cell line upon WDR5 silencing are reported. Blue and orange colors indicate down- and upregulated genes, respectively. **d** Representative Gene Ontology (GO) terms enriched in DEGs of PDXs are shown. For complete list refer to Supplementary Table S3. **e** Hierarchical clustering (Euclidean distance, complete linkage) of log2FC of expression levels (expressed as *z*-score values) of genes involved in enriched functions due to WDR5 silencing in five different PDXs. Listed genes are included in cell cycle progression (Cell division, G1/S transition), epithelial-to-mesenchymal transition (EMT), TGFβ1 signaling, wound healing, cell migration, and cell shape (i.e., actin binding and membrane raft) (see text). Log2FC of each gene was calculated as ratio of gene expression of shWDR5 to shLuc (control) PDX samples (done in triplicate)

A direct transcriptional regulation of tumorigenesis by WDR5 on its targets has been reported in leukemia and bladder cancer [13, 25]. To analyze its role in breast cancer, we evaluated the transcriptional profile by RNA-seq of control (shLuc) or shWDR5 cells in two TN and three LB PDXs (Additional file 2: Table S3; Fig. 2a) and in MCF10DCIS cell line (Additional file 1: Figure S2A). For each PDX, we performed pairwise analysis to identify differentially expressed genes (DEGs). In order to exclude individual specificities, due to the intra- and inter-tumor heterogeneity of BC patients [29], we considered a set of 253 DEGs (161 down- and 92 upregulated) in common between at least 2 PDXs (Additional file 2: Table S3). Among these, 99 down- and 40 upregulated genes were recapitulated in MCF10DCIS (Fig. 2c), identifying a common regulatory trait upon WDR5 silencing. Fourteen out of these genes, and WDR5, were randomly chosen and validated by RT-PCR (Additional file 2: Table S4) in MCF10DCIS and, those defined as DEGs, also in two representative PDXs (MBC26 and MBC7) (Additional file 1: Figure S2B).

In parallel, we assessed H3K4me3 abundance in MCF10DCIS cells (Additional file 2: Table S3), and we observed a global reduction of three-methylation intensity at the promoter level of the expressed genes (± 1500 bp from transcription start site—TSS) in shWDR5 with respect to shLuc (*P* = 3.6E−62) (Additional file 1: Figure S2C). Specifically, we observed a statistically significant reduction in H3K4me3 signal at the promoter of the downregulated (*P* = 1.3E-38), but not of upregulated genes (Additional file 1: Figure S2C). Overall, these data confirmed that WDR5 knockdown reduced target genes transcription and H3K4 three-methylation, associated with active and poised TSS, as shown for representative genes in Additional file 1: Figure S2D.

In order to deeply investigate the involvement of WDR5 in downstream gene regulation, we performed Gene Ontology (GO) analysis of DEGs in PDXs as shown in Fig. 2d. Top-ranking categories included cellular functions that are known to be regulated by WDR5 (i.e., cell division and G1/S transition) [13, 15–18]. Moreover, EMT, cell migration and wound healing, actin cytoskeleton rearrangements, and TGFβ regulation functions were significantly represented after WDR5 knockdown. We analyzed the expression of the most representative genes in the aforementioned enriched cell functions and found that they are transcriptionally regulated, though at different levels, in all PDXs (Fig. 2e). Typically, genes with a role in G1/S transition or cell division (i.e., CCND1, BCAT1, UBE2C, DKC1, CCT4, CCT7) were down-modulated in shWDR5 cells, supporting the role of WDR5 in cell cycle progression. We focused our attention on functions which are consistently associated with metastasis in human tumors [4, 7] and possibly open interesting therapeutic applications in a metastatic setting. Among these, the TGFβ pathway activation is commonly associated with the malignant phenotype of cancer cells [34], as well as with metastasis formation of high-grade BC tumors and BC invasiveness [35]. Indeed, TGFβ1 targets (i.e., FURIN, LTBP2, PARD6A, GCNT2, TGFβR2), as well as other EMT regulators (i.e., SNAI2, ZEB1/2, and TWIST1) were down-modulated in the majority of the shWDR5 PDXs (Fig. 2e). Analogously, genes involved in wound healing and cell migration (i.e., SERPINE1, HMGCR, IGFBP5, and S100A14) or reported as prognostic factors in breast, ovarian, gastric, and lung cancers (i.e., MAPRE1, HSPA1B, and PPME1) [36–38] were negatively modulated. Instead, genes responsible of actin remodeling (i.e., CFL2, CORO1C, FSCN1) and membrane raft (i.e., MAL, BIRC3, PLLP, TNF) were upregulated in WDR5 knockdown condition due to the increased cell polarization and cell-cell adhesion (Fig. 2e). Representative genes with a role in EMT were validated by RT-PCR (dots highlighted in blue), as reported in Additional file 1: Figure S2B.

To extend this observation to a larger cohort of BC patients, WDR5-transcriptionally regulated genes highlighted in Fig. 2e were compared with publicly available gene expression data in cBioportal for Cancer Genomics (https://www.cbioportal.org). Expression analysis showed that the majority of WDR5 targets were overexpressed (*z*-score ≥ + 2) (*n* = 1093 breast cancer patients) (Additional file 1: Figure S3A). Then, we evaluated the expression pattern of these genes in comparison with WDR5 and we found a significant co-overexpression of WDR5 with genes associated with EMT and unfavorable prognosis, such as GCNT2, PPME1, MAPRE1, SNAI2, ZEB2, and S100A14 [39, 40], and tumor growth (UBE2C [41]) (Fisher’s exact test; *P* ≤ 0.05) (Additional file 1: Figure S3B). Collectively, these observations suggest that, as proposed in our model, WDR5 and its targets can be dysregulated in BC patients, likely driving tumorigenic events. Finally, these data confirm that WDR5 regulates a large set of genes affecting both proliferation and EMT in BC.

### WDR5 expression is crucial to sustain the mesenchymal phenotype in breast cancer

**Fig. 3:**
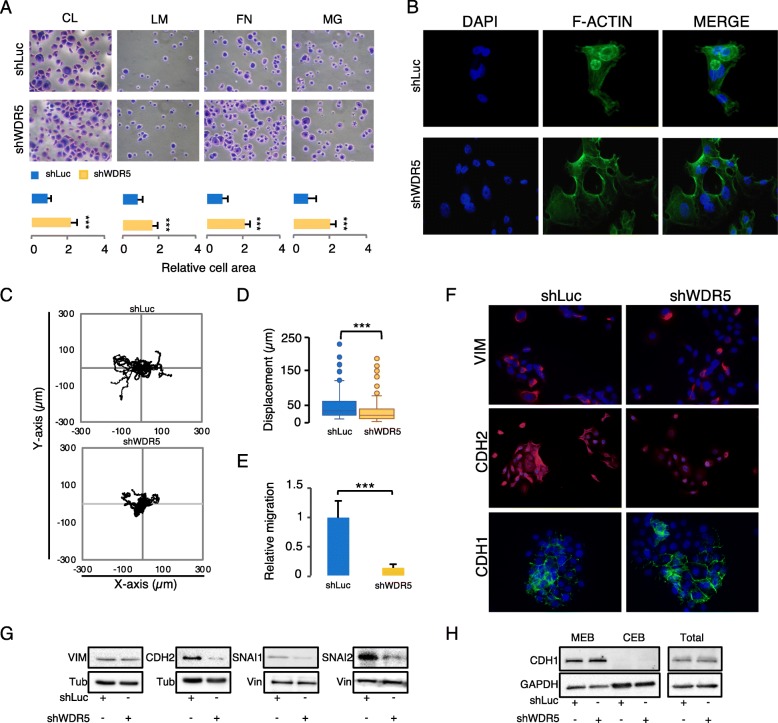
WDR5 silencing inhibits EMT. **a** Adhesion to a panel of substrates (collagen—CL, laminin—LM, fibronectin—FN, matrigel—MG) in shLuc or shWDR5 MCF10DCIS cells was considered. Cells were stained by crystal violet and adhesion property was calculated by ImageJ and expressed as ratio of relative cells area of shWDR5 versus shLuc in biological triplicates. Statistical significances among areas were calculated by applying an unpaired Student *t* test (***P < 0.001). **b** Morphological changes were evaluated by actin cytoskeleton staining of F-actin in MCF10DCIS cells due to WDR5 silencing by immunofluorescence technique (*n* = 3). Images show F-actin (green), DAPI (blue), and merged staining of shLuc and shWDR5 cells. **c** Trajectory plots were obtained by in vitro random migration assay, performed in MCF10DCIS cells. ShLuc and shWDR5 conditions were analyzed by time-lapse microscopy (*n* = 180 cells in shLuc and *n* = 209 cells in shWDR5) and data acquired every 10 min over a 24-h time course. **d** Box plot represents the displacement (μm) of cells as accumulated distance. Significant differences among groups were analyzed by an unpaired Student *t* test (***P < 0.001). **e** Effect of WDR5 silencing was evaluated in terms of in vitro FBS-directed cell migration. Images were compared to quantify the percentage of cell migration (mean ± SD; *n* = 3). Significant differences among groups were calculated by applying an unpaired Student *t* test (***P < 0.001). **f**–**h** Effect of WDR5 silencing on the expression of EMT markers were evaluated by immunofluorescence (**f**) or western blot (**g**) in MCF10DCIS cells. Vimentin (VIM), N-Cadherin (CDH2), and SNAI1 and SNAI2 total protein expression was detected in shLuc and shWDR5 cells. Tubulin (Tub) or Vinculin (Vin) were used as normalizers, according to the molecular weight of proteins analyzed. **h** E-Cadherin (CDH1) level was detected by western blot in membranous (MEB), cytoplasmic (CEB), and total protein lysates. GAPDH was used to assess the amount of total protein lysates in the two samples and as positive control of CEB

Among different hypothesis taking into consideration inherited or acquired traits leading to metastatic events, a large body of literature sustains that the coordinated transcriptional control of cell adhesion, cell shape, migration, and EMT is required to support both invasion and metastasis [2, 4, 10]. We speculate that WDR5 may exert this governance in BC. The adhesion assay performed in control- and shWDR5-MCF10DCIS cells on a panel of extracellular matrices (collagen, laminin, fibronectin, matrigel) showed that cell adhesion was increased in shWDR5 cells both in terms of cell number and cell area (Fig. 3a and Additional file 1: Figure S4A), thus suggesting a tighter cell anchoring to the matrix. Filamentous actin (F-actin), detected by immunofluorescence (IF), was assembled into stress fibers in MCF10DCIS control cells, while in shWDR5 cells predominantly organized in cortical bundles tightly associated with cell-cell adhesions (Fig. 3b), more reminiscent of an epithelial-like phenotype. In order to properly analyze the effects of WDR5 on live cell motility, we performed an in vitro time-lapse random migration assay in MCF10DCIS. WDR5 silencing induced a significant reduction of cell motility with respect to the control (shLuc) (Fig. 3c), quantified as displacement (μm) in the box plots (about 40%) (***: *p* value < 0.001) (Fig. 3d), suggesting that WDR5 regulates breast cancer cell motility, as previously observed in other systems [42]. Reduction of motility was also associated with depletion of FBS-directed migration in MCF10DCIS and PDX cells (Fig. 3e and Additional file 1: Figure S4B), as well as inhibition of wound healing capacity (Additional file 1: Figure S4C) due to WDR5 silencing.

We then characterized the response to WDR5 silencing in terms of mesenchymal and epithelial marker regulation. CDH2, SNAI1, and SNAI2 protein levels were reduced, as shown by IF (Fig. 3f) or western blot (Fig. 3g), although Vimentin (VIM) and Cadherin 1 (CDH1) were only partially affected by WDR5 knockdown. We also evaluated the membranous (MEB), cytoplasmic (CEB), and total protein levels of CDH1 in WDR5 knocked -down cells (Fig. 3h). Following cellular fractionation, CDH1 staining was detected only in MEB, without significant differences in total protein levels between shLuc and shWDR5 cells. These data indicate that CDH1 levels are fully ascribable to the membranous component and that CDH1 is not primarily involved in EMT reversion due to WDR5 silencing. Conversely, overexpression of WDR5 caused changes in cell morphology, leading to an elongated spindle shape of the cells (Additional file 1: Figure S4D), accompanied by changes in gene expression reflecting the mesenchymal scenario (Additional file 1: Figure S4E). These observations suggest that WDR5 is accountable of maintaining an aggressive and metastatic phenotype and that its inhibition determines a partial mesenchymal reversion, thus driving cells toward an epithelial trait.

### WDR5 induces breast cancer metastasis

**Fig. 4:**
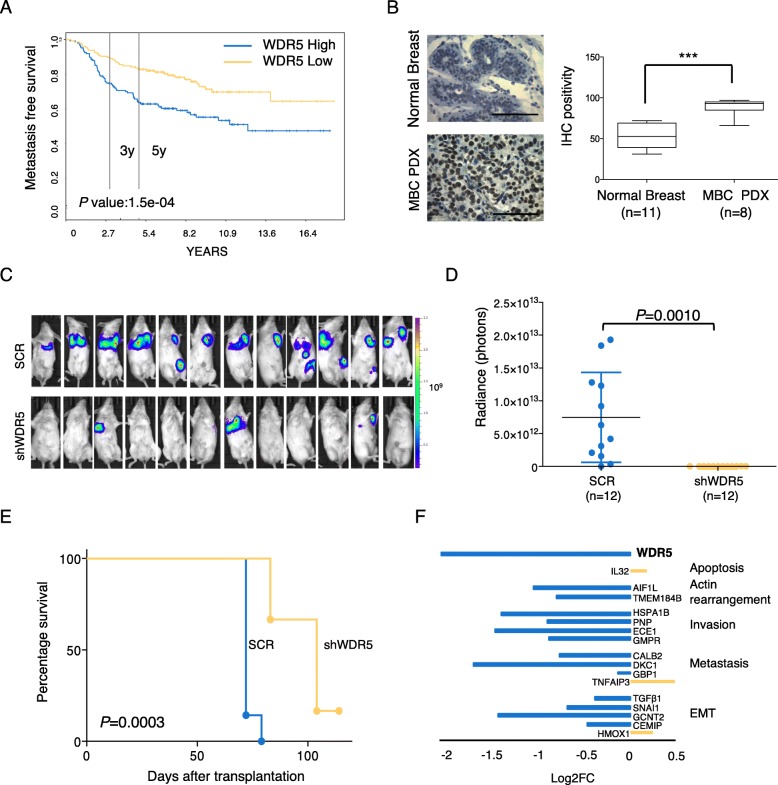
WDR5 silencing significantly reduced breast cancer metastasis. **a**) Metastasis free survival in breast cancer patients (*n* = 295) was calculated in WDR5-low and WDR5-high groups divided at median of gene expression. Statistic comparison was performed using log-rank test. **b**) Representative IHC staining of WDR5 expression in normal breast tissues (*n* = 11) and Metastatic Breast Cancer (MBC) PDXs (*n* = 8) is reported. Scale bar 100 μm. Box plots represent WDR5 expression quantified by using Fiji tools for DAB positivity. Statistical significance was determined using an unpaired Student *t* test (***: *P* < 0.001). **c**) Effect of WDR5 silencing on metastasis formation in vivo in MDA-MB-231. Luciferase-transduced cells were infected to silence WDR5 or a control vector (SCR) and then transplanted in NSG mice (*n* = 12 *per* group). Metastases at distant organs were detected by using bioluminescence technique in vivo after resection of primary tumors as reported in Fig. **d**) Dot plots represent luminescence expressed in radiance of photons detected at metastatic sites in SCR and shWDR5 groups. Values were calculated by IVIS Illumina Software and compared by applying an unpaired Student *t* test (*P* = 0.0010). **e**) Differences among SCR and shWDR5 groups in terms of percentage of survival were calculated by using Log-rank test (*n* = 7; *P* = 0.0003). **f**) RT-PCR was performed in SCR and shWDR5 MDA-MB-231 cells. Bars represent Log2FC of ∆∆CT values of shWDR5 to SCR of WDR5-target genes, classified on the basis of their biological functions

In order to correlate WDR5 expression and the metastatic process, by using a second independent data set [31], we evaluated WDR5 expression and metastasis-free survival (MFS) association in a cohort of BC patients (*n* = 295; WDR5 high expression = 148; WDR5 low expression = 147). Higher WDR5 expression was associated with worse MFS (Fig. 4a), confirming the prognostic role of WDR5 in the metastatic event. Moreover, MFS analysis adjusted for ER and therapeutic (endocrine or chemotherapy) status showed that patient outcome was independent of these factors (Additional file 1: Figure S5A). In addition, we compared WDR5 protein levels of normal breast with MBC PDX tissues by IHC (Fig. 4b) and of MCF10DCIS and MDA-MB-231 BC cell lines with a non-transformed counterpart, MCF10A, by western blot (Additional file 1: Figure S5B). In both cases, BC samples, and remarkably the metastatic ones, showed increased WDR5 levels with respect to normal/non-transformed ones, thus paralleling observation on metastatic BC patients. In order to validate this observation, we used MDA-MB-231 cells that are able to maintain the metastatic tropism and represent a successful model for studying BC metastasis. We compared the metastatic ability of SCR and shWDR5 MDA-MB-231 cells by performing a functional in vivo assay that recapitulated the phases of metastatic cascade from primary tumor to metastasis formation. Cells were transplanted in the fourth mammary gland of NSG mice (*n* = 12); tumors were resected at the same volume, both in control and WDR5-silenced groups; and metastases at distant organs were detected (Additional file 1: Figure S5C). MDA-MB-231 metastasized to lung and axillary lymph nodes in SCR (Fig. 4c, upper panel), while WDR5 silencing totally inhibited (9/12), or significantly reduced (3/12), the number and size of the metastatic foci (Fig. 4c, lower panel), as confirmed by the highly significant difference in luminescence (expressed as radiance) among the two groups (*P* = 0.001; Fig. 4d). In addition, mice in which WDR5 was silenced showed a significant survival (*P* = 0.0003) with respect to the control (Fig. 4e). Corresponding to the in vivo results, WDR5 silencing led to a consistent reduction of MDA-MB-231 in vitro FBS-directed cell migration (Additional file 1: Figure S5D), wound healing (Additional file 1: Figure S5E), and random migration assays (Additional file 1: Figure S5F), confirming that WDR5 impacts on the ability of BC cells to migrate and metastasize to distant organs and that its expression is predictive of metastatic disease*.* Then, we confirmed by RT-PCR that the expression of WDR5-downstream genes involved in EMT and metastatic phenotype was also modulated in MDA-MB-231 upon WDR5 silencing (Fig. 4f), thus suggesting that WDR5 inhibition may be a useful approach to induce the reversion of the mesenchymal trait in a metastatic setting.

### WDR5 regulates TGFβ1 activation in breast cancer

**Fig. 5:**
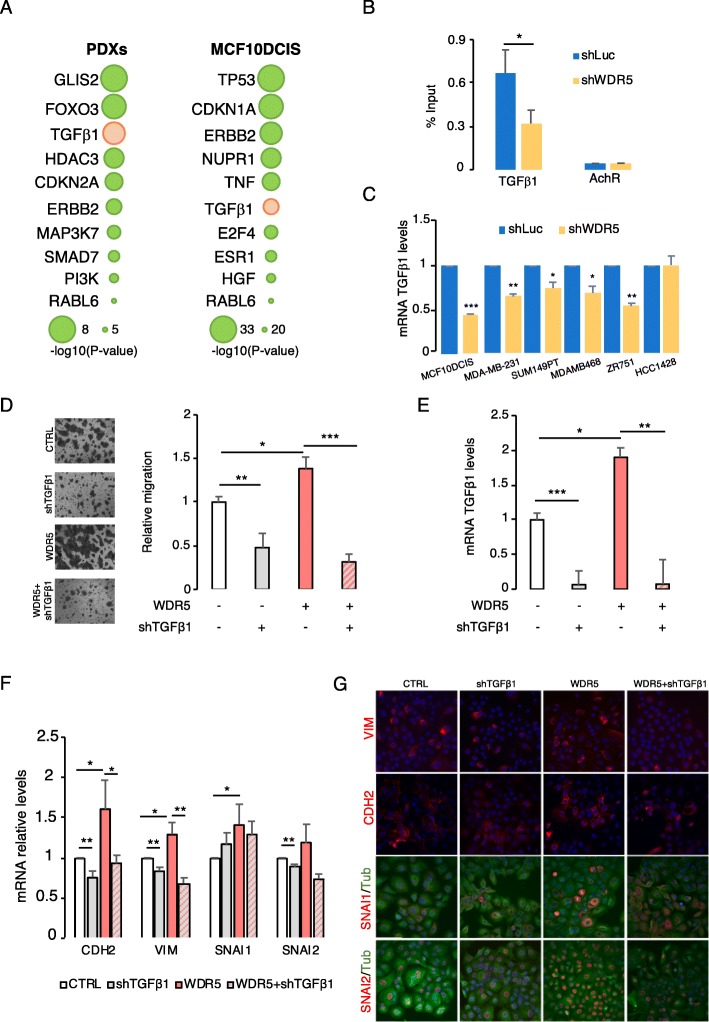
WDR5 regulates TGFβ1 in breast cancer. **a** Ingenuity pathway analysis was used for upstream pathway evaluation. Representative regulators of DEGs in PDXs and MCF10DCIS are shown. For complete list, refer to Supplementary Table S3. TGFβ1 is reported as one of the most significative upstream regulators in all samples. **b** Quantitative chromatin immunoprecipitation (qChIP) was used to analyze binding of WDR5 to TGFβ1 promoter in MCF10DCIS cells. qChIP was performed on shLuc and shWDR5 cells and values expressed as percentage of input chromatin (mean ± SD; *n* = 2; *P < 0.05). **c** RT-PCR was performed to evaluate TGFβ1 mRNA level reduction upon WDR5 silencing in MCF10DCIS, MDA-MB-231, and other BC cell lines (2 TN—SUM149PT and MDAMB468—and 2 LB—ZR751 and HCC1428). Statistical significance among shLuc and shWDR5 groups was calculated by applying a Student *t* test (*P < 0.05; **P < 0.01; ***P < 0.001). **d**–**g** MCF10DCIS cells were infected to silence TGFβ1 (shTGFβ1) in presence (+) or absence (−) of WDR5 overexpression. **d** Relative migration was evaluated in the indicated conditions and representative images reported. Statistical significance among groups was calculated by applying one-way ANOVA followed by the Bonferroni post test (*P < 0.05; **P < 0.01; ***P < 0.001). **e** TGFβ1 mRNA levels were evaluated by RT-PCR in presence (+) or absence (−) of WDR5 overexpression. Statistical significance among groups was calculated by applying an unpaired Student *t* test (*P < 0.05; **P < 0.01; ***P < 0.001). **f**, **g** EMT markers were evaluated by RT-PCR (**f**) and immunofluorescence (IF) techniques (**g**) in the indicated conditions. Statistical significance among groups was calculated by applying an unpaired Student *t* test (*P < 0.05; **P < 0.01). Nuclei are stained in blue (DAPI), cell morphology assessed by tubulin staining (in green), VIM, CDH2, SNAI1, and SNAI2 are labeled in red (× 40 magnification)

**Fig. 6:**
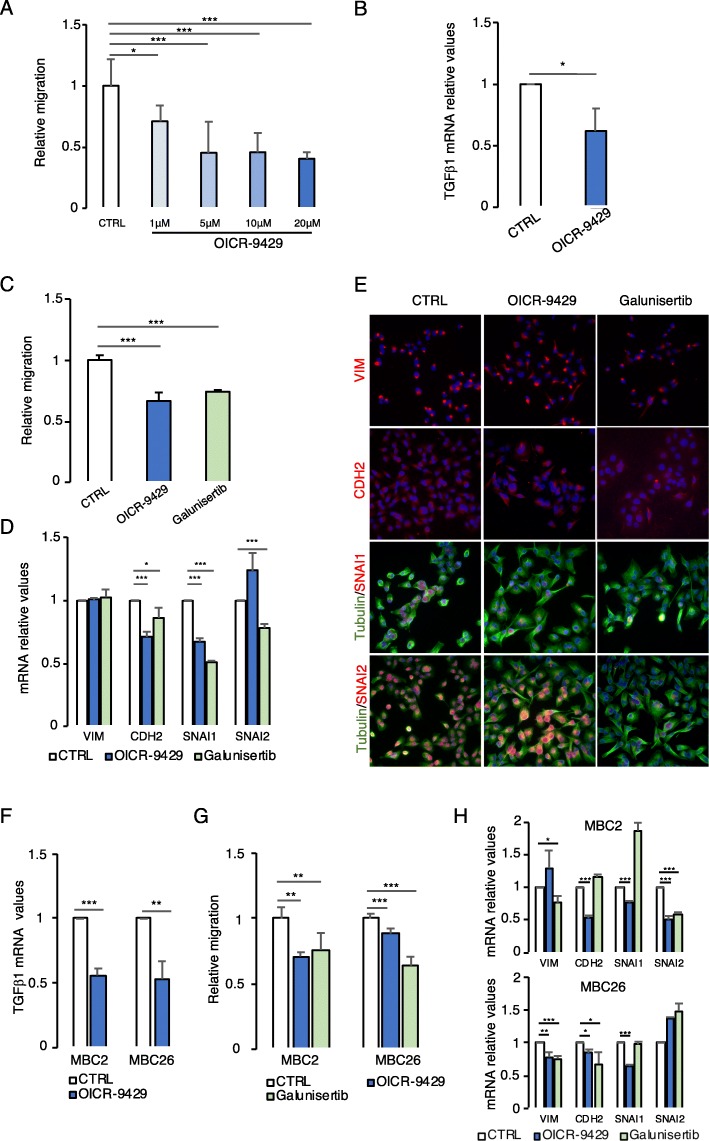
WDR5-TGFβ1 inhibition reduces cell migration and EMT gene expression in breast cancer. **a** Effect of WDR5 inhibitor OICR-9429 at increasing concentrations (1 μM, 5 μM, 10 μM, 20 μM, 3 days of treatment) was evaluated in MDA-MB-231 cell line. Cells were plated on transwells to test in vitro cell migration, stained with crystal violet, and quantified using ImageJ analysis. Histograms represent relative cell migration expressed as mean ± SD of a biological triplicate. Differences among groups were calculated by applying one-way ANOVA and Dunnett post test (*P < 0.05; ***P < 0.001). **b** TGFβ1 mRNA levels (mean ± SD of *n* = 3 experiments) were evaluated by RT-PCR upon OICR-9429 treatment (20 μM) in MDA-MB-231 cells. Statistical differences were calculated by applying a Student *t* test (*P < 0.05). **c** MDA-MB-231 cells were treated with OICR-9429 (20 μM) and galunisertib (10 μM—TGFβ1 inhibitor) for 3 days and cells were plated on transwells for the evaluation of in vitro cell migration. Differences among groups were evaluated by applying one-way ANOVA and Dunnett post test (***P < 0.001). **d**, **e** VIM, CDH2, SNAI1, and SNAI2 expression was evaluated by RT-PCR (**d**) and immunofluorescence (**e**) upon OICR-9429 (20 μM) and galunisertib (10 μM) treatments. Differences in mRNA levels among groups were evaluated by applying an unpaired Student *t* test (*P < 0.05; ***P < 0.001). For immunofluorescence, DAPI (in blue) was used for nuclei staining and tubulin (in green) for cell morphology staining. VIM, CDH2, SNAI1, and SNAI2 are labeled in red. **f** Effects of OICR-9429 (20 μM) on TGFβ1 mRNA levels were evaluated in one TN (MBC2) and one LB (MBC26) PDXs. Statistical differences among groups were calculated by applying a Student *t* test (**P < 0.01; ***P < 0.001). **g** Effects of OICR-9429 (20 μM) and galunisertib (10 μM) were evaluated in terms of in vitro cell migration after 3 days of treatment in MBC2 and MBC26 PDX cells (mean ± SD of *n* = 3 experiments). Differences among groups were evaluated by applying one-way ANOVA and Dunnett post test (**P < 0.01; ***P < 0.001). **h** mRNA levels of VIM, CDH2, SNAI1, and SNAI2 were evaluated by RT-PCR due to aforementioned treatments in MBC2 and MBC26 PDX cells. Statistical significances among groups were calculated by applying a Student *t* test (*P < 0.05; **P < 0.01; ***P < 0.001)

As suggested by the analysis on RNA-seq profile, WDR5 transcriptionally modulates genes involved in the EMT. In order to deeply investigate the direct interaction of WDR5 and its targets, we first performed an upstream analysis of the DEGs in PDXs and in MCF10DCIS cells. Ingenuity pathway analysis (IPA) confirmed the putative involvement of genes that regulate EMT (HDAC3, ERBB2, SMAD7) [8, 28, 43] or drive tumor progression and metastatic dissemination (i.e., TP53, TNF, ESR1, HGF*,* PI3K) in BC. Interestingly, TGFβ1 resulted as one of the top-ranked upstream genes among PDXs and MCF10DCIS (Fig. 5a). Then, chromatin immunoprecipitation (ChIP) and sequencing was performed to analyze the genome-wide WDR5 occupancy in MCF10DCIS cells (Additional file 2: Table S3). We found that around 80% (4413/5794) of the WDR5 peaks were located in close proximity to the TSS (± 3000 bp), confirming that WDR5 regulates its targets mainly at the promoter level. As expected, some already known interactors of WDR5 (i.e., TWIST1, ERBB2, LYN, and RBM22) [14, 22, 25] are regulated on their promoters (Table S3), thus validating our analysis. When RNA-seq and WDR5 peaks were intersected, we found 364 WDR5 peaks overlapping with downregulated and 189 corresponding with upregulated genes (Additional file 1: Figure S6A). Among these, WDR5 bound TGFβ1 promoter, as confirmed by the quantitative chromatin immunoprecipitation (qChIP) performed in shLuc and shWDR5 MCF10DCIS cells (Fig. 5b). Indeed, WDR5 knockdown inhibited TGFβ1 transcription in MCF10DCIS (Fig. 6b and Fig. 5c), as well as in MDA-MB-231 and in diverse LB and TN BC cell lines (Fig. 5c). Since TGFβ1 is a potent inducer of the EMT in mammary cells [35], we hypothesized that WDR5 may regulate TGFβ1 to orchestrate the observed phenotype. In order to confirm this observation, we silenced TGFβ1 (shTGFβ1) in presence (+) or absence (−) of WDR5 overexpression in MCF10DCIS cells. First, we observed that TGFβ1 knockdown significantly reduced cell migration (Fig. 5d) and wound healing (Additional file 1: Figure S6C), as shown with WDR5 silencing. Strikingly, shTGFβ1 abrogated MCF10DCIS migration induced by WDR5 overexpression (Fig. 5d), that in turn increased TGFβ1 mRNA levels (Fig. 5e). We also evaluated in the same conditions the mesenchymal markers known to be regulated by WDR5 (Fig. 3f–h), and we confirmed that TGFβ1 silencing was able to interfere with the WDR5-induced mesenchymal phenotype, by inhibiting EMT genes at the RNA (RT-PCR, Fig. 5f) and protein (IF, Fig. 5g) levels. Overall, these data confirm that TGFβ1 is responsible, at least in part, for WDR5 phenotype and that the inhibition of the WDR5-TGFβ1 axis could be envisioned as a therapeutic strategy to inhibit EMT.

### Drug targeting of WDR5-TGFβ1 axis reduces breast cancer aggressiveness

WDR5 inhibitor OICR-9429 has been successfully used to reduce tumorigenesis of leukemia, pancreatic cancer, and neuroblastoma [15, 17, 44]. Moreover, recently, an in vivo approach by monobody against WDR5 has been tested in a leukemia background [45]. In addition, the inhibitors of TGFβ1 receptors have been proposed as new anti-metastatic therapy [46], such as galunisertib (LY2157299), that has entered phase II/III clinical trials in pancreatic cancer, glioblastoma, hepatocellular carcinoma, and myelodysplastic syndrome [47].

We first used MDA-MB-231 and MCF10DCIS to test the efficacy of OICR-9429 in regulating cell migration. OICR-9429 significantly reduced BC cell line migration in a dose-dependent manner (Fig. 6a and Additional file 1: S7A), while it did not affect the migration of the MCF10A non-transformed cells (Additional file 1: Figure S7B), suggesting that WDR5 inhibition may exert specific effects on tumor cells. We confirmed that OICR-9429 treatment (20 μM), as WDR5 silencing, resulted in TGFβ1 mRNA level reduction in MDA-MB-231 (Fig. 6b) and MCF10DCIS (Additional file 1: Figure S7C). Then, we confirmed that galunisertib (10 μM) significantly reduced MDA-MB-231 and MCF10DCIS cell migration (Fig. 6c and Additional file 1: Figure S7D). In MDA-MB-231 cells, drug administration exerted an analogous effect on the most relevant EMT markers, by reducing CDH2 and SNAI1, without affecting VIM, in terms of relative mRNA levels verified by RT-PCR (Fig. 6d) and protein expression by IF (Fig. 6e). In MCF10DCIS, CDH2 and SNAI2 were significantly reduced due to both treatments (Additional file 1: Figure S7E), while SNAI1, although down-modulated, reached significance only in galunisertib treatment. Moreover, drug administration exerted a drastic change of MCF10DCIS cell shape that assumes a more cobblestone-like morphology with epithelial features, as shown by tubulin staining (Additional file 1: Figure S7F).

Finally, in two different PDXs (one LB: MBC26 and one TN: MBC2), OICR-9429 administration (20 μM) significantly reduced TGFβ1 mRNA levels (Fig. 6f), migratory potential (Fig. 6g), and expression of mesenchymal genes (Fig. 6h). These effects were recapitulated, at least in part, by galunisertib administration (10 μM). In MBC26, drug treatment caused a slight increase in SNAI2 expression that can be justified by the fact that EMT and its reversion may generate a mixed population of cells, possibly accounting for a partial EMT phenotype. SNAI1 is anyway sufficient to ensure EMT, as reported elsewhere in BC cells [48]. Overall, these data confirm that drugs targeting WDR5-TGFβ1 axis significantly inhibit EMT in BC. It can therefore be speculated that, by suppressing mesenchymal features of cancer cells, these drugs may have a strong potential of reducing BC metastasis.

### WDR5-TGFβ1 inhibition restores drug sensitivity

The transition from a mesenchymal to an epithelial state with drug treatment can be used to enhance lethality and eradicate epithelial cells, as well as a strategy to sensitize cells to chemotherapy [3]. We used MBC2 and MBC26 PDXs to test the effect of WDR5 inhibition on lethality in combination with paclitaxel (PTX), indicated for first-line therapy in TN breast cancer and later-line therapy in ER+ metastatic breast cancer [49].

**Fig. 7:**
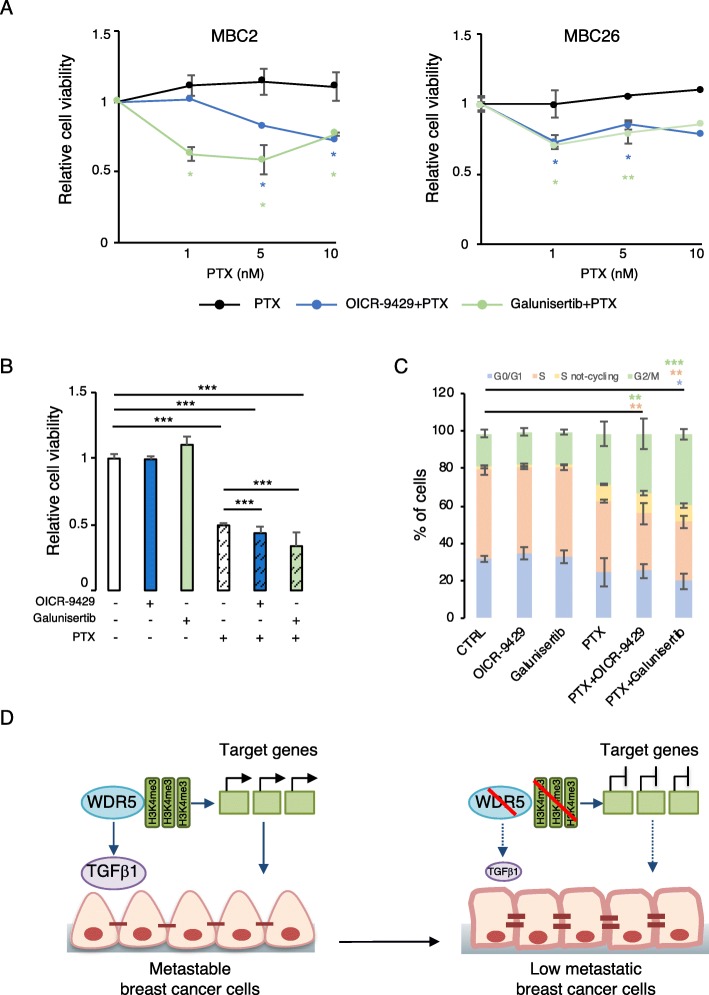
WDR5 inhibitor sensitizes breast cancer cells to paclitaxel. **a** One TN (MBC2) and one LB (MBC26) PDXs were treated for 3 days with diverse concentrations of paclitaxel (PTX) (1 nM, 5 nM, and 10 nM) alone or in combination with OICR-9429 (20 μM) or galunisertib (10 μM). Cell viability is reported as ratio of the control group (vehicle). Differences among indicated concentrations were calculated by applying a Student *t* test (mean ± SD of *n* = 3 experiments; *P < 0.05; **P < 0.01). Asterisk colors are associated with corresponding treatment. **b** MDA-MB-231 cells were treated for 3 days with PTX (10 nM) upon OICR-9429 (20 μM) or galunisertib (10 μM) treatments. Cell viability of each condition was expressed as ratio to the control group (vehicle) and significant differences among groups calculated by an unpaired Student *t* test (mean ± SD of *n* = 3 biological replicas; ***P < 0.001). **c** MDA-MB-231 cells were treated as above and analyzed by flow cytometry for bromodeoxyhuridine (BrDU) and propidium iodide (PI) content. Percentage (%) of cell population in each phase of cell cycle (G0/G1, S, S-not cycling and G2/M) is represented for each condition. Statistical significance was calculated by applying two-way ANOVA followed by the Bonferroni post test for multiple comparisons (*P < 0.05; **P < 0.01; ***P < 0.001). Colors of the asterisks are associated with the phase of the cell cycle in which the differences were detected. **d** Schematic representation of WDR5 mechanism of action in breast cancer for the maintenance of cells hovering from mesenchymal to epithelial-like phenotype. TGFβ1 is directly regulated by WDR5 and largely participates to this governance so effectively that inhibition of WDR5-TGFβ1 axis switches cell state from metastable to low metastatic and reduces breast cancer progression

As previously shown, MBC2 and MBC26 PDXs showed resistance to different doses of PTX [29] (Fig. 7a). Strikingly, OICR-9429 or galunisertib, that did not reduce proliferation per se in both PDXs (Additional file 1: Figure S8A), sensitized cells to PTX reducing cell viability in combinatorial treatments (Fig. 7a). As observed in MDA-MB-231 cells, administration of OICR-9429 or galunisertib and PTX, at the half maximal inhibitory concentration (10 nM), additionally reduced about 10–15% cell viability (Fig. 7b), marked by cleavage of PARP, increased DNA damage (through the phosphorylation of H2AX), and slight reduction of PCNA (Additional file 1: Figure S8B). Since PTX treatment results in the disruption of the microtubule dynamics, which is essential during cell division, we performed analysis of the bromodeoxyuridine (BrDu) and propidium iodide (PI) content in MDA-MB-231 in the aforementioned conditions. MDA-MB-231 cells treated with PTX were unable to regularly proceed through the cell cycle, as suggested by an increase of the percentage of cells in the G2-M phase, and a concomitant reduction of S and G1 phases. Although OICR-9429 or galunisertib did not alter cell cycle progression, the combinatorial administration of PTX further increased the block of the cell cycle (Fig. 7c and Additional file 1: Figure S8C), thus suggesting that inhibition of WDR5 or TGFβ1 signaling can potentiate the activity of chemotherapy. In addition, both in PTX and combinatorial treatments, a not-cycling S phase population was detected, confirming that a substantial percentage of the cells was not proliferating (Fig. 7c and Additional file 1: Figure S8C).

Overall, these data indicate that inhibiting WDR5, or its target TGFβ1, is an effective strategy to sensitize drug-resistant cells to chemotherapy, as found for ZEB1, TWIST, and SNAIL [50–52], thus suggesting a rational combination to restrict BC progression.

## Discussion

It is largely recognized that the dysregulation of chromatin remodelers is often associated with, or drives the development of, human cancers [10]. Despite this notion, the molecular basis of such connection is still a matter of debate. Moreover, epigenetic modifications may underlie cancer-specific phenotypes and represent molecular vulnerabilities that can be targeted in cancer therapy.

In this study, we report that WDR5, the core subunit of methyltransferase complex, is an essential gene for breast cancer progression since its inhibition is associated with reduction of tumorigenesis and metastasis. We demonstrate that loss of WDR5 transcriptionally represses its target genes and uncouple TGFβ1 pathway and EMT, thus inducing a switch from a mesenchymal-like phenotype toward an epithelial status (Fig. 7d) in BC cells. Then, we suggest WDR5 inhibition as a therapeutic strategy to hit the EMT network, reduce metastasis, and sensitize breast cancer cells to chemotherapy.

Consistent with the prognostic role of WDR5 [24–27], we associated its expression with breast cancer progression and aggressiveness, by making use of a panel of cell lines and PDX models of metastatic tumors, the latter representing patients resistant to different lines of therapy. In agreement with this observation, we showed that WDR5 inhibition was effective in reducing tumor growth in all LB and TN breast cancers samples, independently by ER status or therapies. In addition, WDR5 loss reduced metastasis dissemination of BC cells in vivo. The ability of tumor cells to metastasize has been frequently correlated with the EMT, since the transition from a well-differentiated epithelial phenotype to an invasive mesenchymal state, whose regulation is under transcriptional and post-transcriptional control, may enhance cell motility and invasiveness [4, 7]. Previous works also suggest that WDR5 plays a critical role in the regulation of tumor cell migration, as well as of invasion in a zebrafish transplantation model [9, 28, 42, 53], and in the control of metastasis formation by inducing EMT in various cancers [21–23]. We speculated that WDR5 could induce EMT in breast cancer and that its inhibition could reverse the mesenchymal phenotype, consistent with known plasticity of this cellular program whereby cells switch from the mesenchymal to the epithelial states and back [2]. By employing gene expression profiling upon WDR5 inhibition in breast cancer, we found that WDR5 transcriptionally regulates gene signatures, typically involved either in proliferation and cell cycle, or in metastasis, EMT, and their correlated functions. Thus, this governance can also be ascribed to epigenetic regulation, since active histone mark (H3K4me3) was found significantly modified at the promoter of differentially expressed genes. We have demonstrated that, beyond the effects on proliferation, WDR5 inhibition drives cells to a partial epithelial status by reducing expression of the main mesenchymal genes and cell motility and migration, and partly restoring the typical features of epithelial-like cells (i.e., polarization and cell-cell adhesion). Our genome-wide binding site analysis showed that WDR5 binds to the TGFβ1 promoter. TGFβ1 signaling and EMT are strictly interconnected in cancer since TGFβ1 controls cell motility and the mesenchymal properties of the cells [7, 54]. We speculated that a similar mechanism is in place in breast cancer and is due to WDR5 regulation, which has a prominent role per se in EMT and metastasis [17, 18, 23], as well as through TGFβ1. We demonstrated that WDR5 inhibition reduced TGFβ1 levels and that TGFβ1 silencing, in turn, is capable of abrogating WDR5-dependent migration, thus confirming that TGFβ1 is crucial (at least in part) in leading WDR5-dependent mesenchymal phenotype. This observation takes into consideration endogenous activation of TGFβ1 and can complement that one reported by Chen and colleagues contemplating TGFβ1 exogenous stimulation [9]. Moreover, the role of TGFβ1 in driving EMT has been predominantly reported for basal-like cells, largely superimposable to the cells of the TN subtype [55]. Our observations suggest that also luminal-like breast cancer is poised to respond to EMT signals and that, concordantly, its inhibition can be a useful method to suppress mesenchymal properties associated to metastasis of cancer cells.

Recently, therapeutic investigations have shown increased interest in the EMT estimation. In fact, diverse clinical trials have included the evaluation of biomarkers of EMT as translational endpoint and diagnostic tool for the detection of circulating tumor cells for advanced breast cancer (Clinicaltrials.gov). Considering the existence of the EMT gradients, evidences indicate that the efficacy of mesenchymal reversal may be cancer type dependent and should be based on a specific therapeutic window to abolish metastasis and enhance drug sensitivity, thus disadvantaging colonization [2]. Although cell differentiation is considered an attractive therapeutic approach to reverse the mesenchymal phenotype, drug discovery platform for EMT switch is still limited. Here, we provide evidences that the WDR5 inhibitor OICR-9429 is able to sensitize breast cancer cells to chemotherapy by reversing the mesenchymal phenotype, overcoming drug resistance, as similarly reported for other epigenetic inhibitors undergoing clinical trials (i.e., Mocetinostat) [3]. Indeed, WDR5 inhibitor sensitizes cells to paclitaxel, revealing a promising combination to eradicate tumor cells in chemo-resistant breast cancer patients.

## Conclusions

In conclusion, we have demonstrated that WDR5 couples EMT and metastatic progression in breast cancer. The inhibitory effects on tumorigenesis and progression due to WDR5 block in PDXs, derived from metastatic patients resistant to therapies, are encouraging for further clinical investigations and suggest a new therapeutic chance for these patients. Finally, our data support WDR5 inhibition as a novel strategy to reverse mesenchymal features and sensitize cancer cells to chemotherapy, thus restricting tumor metastasis in breast cancer.

## Supplementary information

**Additional file 1: Figure S1.** Effect of WDR5 silencing in Luminal B and Triple Negative breast cancer cell lines. **Figure S2.** WDR5 transcriptionally regulates its targets. **Figure S3.** WDR5-target genes are regulated in a larger cohort of patients. **Figure S4.** WDR5 regulates adhesion, migration and polarization of breast cancer cells. **Figure S5.** WDR5 silencing significantly inhibits migration of MDA-MB-231 breast cancer cells. **Figure S6.** TGFβ1 is transcriptionally regulated by WDR5. **Figure S7.** WDR5 inhibition reduces migration of breast cancer cells but not of non-transformed MCF10A breast cells. **Figure S8.** WDR5-TGFβ1 inhibition combined with Paclitaxel causes cell cycle block in MDA-MB-231 breast cancer cells.

**Additional file 2.** **Table S1.** Patients' tumor characterization for site of metastasis, clinical markers and treatments at the moment of relapse is reported. MBC: metastatic breast cancer. ER: Estrogen Receptor. PgR: Progesterone Receptor. LB: Luminal B. TN: Triple Negative. HER2 negative (-). ViFuP: Vinorelbine/ Fluorouracil/ Cisplatin. BEXE: Bevacizumab/ Capecitabine/ Cyclophosphamide/ Erlotinib. Table S2. Biological characterization for the main clinical markers of each patient (PT) and the corresponding PDX at passage in mice used for experimental purpose. MBC: metastatic breast cancer. LB: Luminal B. TN: Triple Negative. Table S3. List of 253 genes from MBC PDXs obtained by in common differentially expressed genes in at least two comparisons is reported. Gene-Ontology analysis by using DAVID tool (version 6.8 Beta) and Ingenuity Pathway Analysis (IPA) for upstream regulators were performed on PDXs. Differentially expressed genes down- and up-regulated in MCF10DCIS breast cancer cell line are shown. IPA for upstream regulators was also performed on MCF10DCIS. H3K4me3 ChIP-seq was performed on MCF10DCIS breast cancer cell line. Reads were mapped to the promoter region (±1500 bp relative to TSS) for annotated transcripts. Significant differential H3K4me3 values are shown. WDR5 genome wide binding sites on MCF10DCIS were assigned to the nearest proximal and distal transcription start sites (TSS)( ± 3kb). Table S4. RT-PCR primer sequences 5'--->3' are reported.

**Additional file 3.** Supplementary methods.

## Data Availability

Data sets are available in the Gene Expression Omnibus (GEO) database under accession number GSE113289. Supplementary information contains supplementary methods and is available at the journal’s website.
