# Supplementary material for: WDR5 inhibition halts metastasis dissemination by repressing the mesenchymal phenotype of breast cancer cells
Source: Breast Cancer Res. 2019 Nov 21;21:123. doi: 10.1186/s13058-019-1216-y (PMC6873410; doi:10.1186/s13058-019-1216-y)

Additional file 1

Supplementary Figure S1. Effect of WDR5 silencing in Luminal B and Triple Negative breast cancer cell lines. A) Immunoblots represent WDR5 silencing efficacy in MCF10DCIS cells. Two shRNAs, in single or in pool, were used to induce WDR5 silencing. Vinculin was used as normalizer. B) MCF10DCIS cells were transduced to over-express WDR5 or a neutral control (SCR). WDR5 over-expression was verified by western blot. Tubulin was used as normalizer. C) Cells over-expressing WDR5 were transplanted in NOD-SCID mice (n=3 *per* group). Pictures showed tumors of the two groups. Tumor volumes were expressed as mean ± SD (cm^3) and significant differences among groups were calculated by using an unpaired Student *t* Test (*: P<0.05). D-E) Six metastatic breast cancer PDXs (D), 2 triple negative (TN – SUM149PT and MDAMB468) and 2 luminal B (LB – HCC1428 and ZR751) breast cancer cell lines (E) were independently infected with the two shRNAs in pool to silence WDR5 or a neutral control (shLuc). Silencing efficacy was evaluated by western blot and vinculin was used as normalizer. F) *In vivo* effect of WDR5 silencing in SUM149PT, MDAMB468, HCC1428 and ZR751. WDR5-silenced cells and corresponding neutral controls were inoculated in the fat pad of immunocompromised mice. Dot plots represent tumors volume in shWDR5 and shLuc cell lines (mean and SD). Statistical significances were calculated by applying an unpaired Student *t* test (*: P<0.05; **: P<0.01; ***: P<0.001).

Supplementary Figure S2. WDR5 transcriptionally regulates its targets. A) Volcano plot shows Differentially Expressed Genes (DEGs) in shWDR5 MCF10DCIS cancer cell line. The log2 expression fold change (FC) is shown on the horizontal axis and -log10 of the FDR is shown on the vertical axis. Up-regulated genes (FDR < 0.05 and log2 FC > 0.6) are

highlighted in orange while down-regulated genes in blue (FDR < 0.05 and log2 FC < -0.6). B) RT-PCR was used to validate 14 out of WDR5 target genes identified as DEGs in RNA-seq analysis. Dot plots represent log2FC values of DEGs in MCF10DCIS and two representative PDXs (MBC26 and MBC7). Pearson correlation (r) was calculated comparing log2FC values obtained from RT-PCR and RNA-seq. Dots highlighted in blue represent genes with a role in EMT and metastasis. C) Box plots show H3K4me3 signal intensity at gene promoters in shLuc and shWDR5 MCF10DCIS cells. Central values represent the median, the bars the 25th and 75th percentile and the dashed lines the lower and upper whiskers. *P* values were obtained from a Wilcoxon rank-sum test (ns: not significant). D) Snapshots show some of the representative genes deregulated in terms of H3K4me3 intensity and RNA upon WDR5 silencing in MCF10DCIS.

Supplementary Figure S3. WDR5-target genes are regulated in a larger cohort of patients. Expression of WDR5 regulated-genes were evaluated in TCGA data set of breast cancer patients (n=1093) in cBioportal. A) Heatmap represents expression (z-score values) of genes from RNA-seq data in each patient. B) Heatmap represents *P*-values, calculated by applying Fisher exact test, of the co-occurrent expression of each indicated gene and WDR5.

Supplementary Figure S4. WDR5 regulates adhesion, migration and polarization of breast cancer cells. A) Cell adhesion of shLuc or shWDR5 MCF10DCIS cells to a panel of substrates (collagen - CL, laminin - LM, fibronectin - FN, matrigel - MG). Cells were stained with crystal violet and percentage of adhesion was calculated by ImageJ and expressed as ratio of relative cell number of shWDR5 versus shLuc of n=3 experiments (mean±SD). Statistical significances among groups were calculated by applying an unpaired Student *t*

test (*: *P*<0.05; **: *P*<0.01). B) *In vitro* migration was analyzed in six MBC PDXs in duplicate. Relative migration (mean ± SD) was calculated by ImageJ analysis of crystal violet stained cells and expressed as ratio of silenced versus control group. Statistical significances were calculated by applying an unpaired Student *t* test (*: *P*<0.05; **: *P*<0.01). C) Wound-healing assay was performed by creating a wound in a cell monolayer of shLuc and shWDR5 MCF10DCIS cells. Images were taken immediately after scratching or at regular intervals until 48h. Dotted lines represent the migration front. Images were used to compare the percentage of cell migration needed to close the wound (mean±SD; n=3). Significant differences among groups were calculated by applying an unpaired Student *t* test (**: P<0.01). D) Changes in cell morphology in WDR5 over-expressed and control (SCR) MCF10DCIS cells at 20X magnification. Inserts show a higher magnification of the selected area. E) mRNA levels of WDR5 and EMT genes in MCF10DCIS cells transduced with control (SCR) or WDR5 over-expressing vectors. RNA levels were estimated by RT-PCR (n=3; mean±SD) and statistically significant differences were calculated by applying an unpaired Student *t* test (*: *P*<0.05).

Supplementary Figure S5. WDR5 silencing significantly inhibits migration of MDA-MB-231 breast cancer cells. A) Metastasis free survival in breast cancer patients (n=295) was calculated in WDR5-low and WDR5-high groups divided at median of gene expression. Association between WDR5 expression and survival was reported with respect to main groups or estrogen (ER) status, or chemotherapy/endocrine therapy (Hazard Ratio: HR, 95% confidence interval: CI). B) Immunoblot represents WDR5 protein levels in MCF10A non-transformed breast cell line, MCF10DCIS and MDA-MB-231 breast cancer cell lines. Relative quantitation of specific signals was performed through normalization to Vinculin. C) MDA-MB-231 cells were used to perform a metastatic assay *in vivo* to compare metastasis

dissemination of scramble (SCR) and shWDR5 cells. Transduced MDA-MB-231 cells were transplanted in the 4th mammary fat pad of NSG mice (n=12 *per* group). Box plots represent volumes (mean ± SD - cm^3) of primary tumors resected in both groups. D) Effect of WDR5 silencing on *in vitro* migration in MDA-MB-231 cell line. Cells were independently infected to silence WDR5 (shWDR5) or a neutral control (shLuc). *In vitro* relative migration values of shWDR5 are reported with respect to control shLuc of n=3 experiments. Statistical significance was calculated by applying an unpaired Student *t* test (***: *P*<0.001). E) Wound-healing assay was performed by creating a wound in a cell monolayer in MDA-MB-231 cells. Images were taken immediately after scratching and at regular intervals until 24h (n=3; mean ± SD). The percentage (%) of cell migration needed to close the wound was quantified by ImageJ analysis and differences among groups calculated by applying a Student *t* test (***: P<0.001). F) Trajectory plots were obtained by random migration assay performed in MDA-MB-231 cells. ShLuc and shWDR5 conditions were analyzed by time-lapse microscopy (n=195 in shLuc cells and n=204 in shWDR5 cells) and data acquired every 10 minutes over a 24 hour time course. Box plot represents the displacement (μm) of cells as accumulated distance. Significant differences among groups were analyzed by unpaired Student *t* test (***: P< 0.001).

Supplementary Figure S6. TGFβ1 is transcriptionally regulated by WDR5. A) Venn diagrams represent intersection of down- or up-regulated genes and WDR5 peaks (±3000 bp region around the annotated transcription start site) in the MCF10DCIS cell line. B) Snapshot represents WDR5 binding peaks identified by genome wide ChIP-seq analysis and RNA signal of TGFβ1 gene in shLuc and shWDR5 MCF10DCIS cells. C) Wound-healing assay was performed by creating a wound in a monolayer of MCF10DCIS cells infected to silence TGFβ1 (shTGFβ1) in presence or absence of WDR5 over-expression.

Representative images were taken immediately after scratching and at 48h in all conditions. Significant differences among groups were calculated at 48h by applying an unpaired Student *t* test (*: $P<0.05$ - mean±SD).

Supplementary Figure S7. WDR5 inhibition reduces migration of breast cancer cells but not of non-transformed MCF10A breast cells. A-B) WDR5 inhibitor OICR-9429 was used at increasing concentrations in MCF10DCIS (A) and MCF10A (B) cells for three days of treatment and plated on transwells for the evaluation of *in vitro* migration. Migrated cells were stained by crystal violet and relative migration expressed as ratio of shWDR5 cells and control cells by ImageJ analysis (n=3; mean±SD). Differences among groups were evaluated by applying one-way ANOVA and Dunnett Post test (*: $P<0.05$; **:$P<0.01$; ns: not significant). C) TGFβ1 mRNA levels were evaluated upon OICR-9429 treatment (20 μM) in MCF10DCIS. Differences among groups were evaluated by applying Student *t* test (*:$P<0.05$). D-F) MCF10DCIS cells were treated with OICR-9429 (20 μM) and Galunisertib (10 μM - TGFβ1 inhibitor) for three days and cells were plated on transwells for the evaluation of *in vitro* cell migration (D). Differences among groups were evaluated by applying one-way ANOVA and Dunnett Post test (***:$P<0.001$). E) VIM, CDH2, SNAI1 and SNAI2 expression was evaluated by RT-PCR in MCF10DCIS cells upon OICR-9429 or Galunisertib treatments in the aforementioned conditions. Differences in mRNA levels among groups were evaluated by Student *t* test (***: $P<0.001$). F) Immunofluorescence for α-Tubulin (in green) was performed in MCF10DCIS cells in the aforementioned conditions. Nuclei were stained by DAPI.

Supplementary Figure S8. WDR5-TGFβ1 inhibition combined with Paclitaxel causes cell cycle block in MDA-MB-231 breast cancer cells. A) MBC2 and MBC26 PDXs were

treated with OICR-9429 (20 μM) and Galunisertib (10 μM) for three days and cell viability was assessed by Cell Titer Glo assay. Percentage of viable cells was expressed as ratio to the control (vehicle). B) MDA-MB-231 cells were treated for three days with PTX (10 nM) upon OICR-9429 or Galunisertib treatment. Western blot of PARP, PCNA and γH2AX is reported for all the indicated combinations. GAPDH was used as normalizer. C) MDA-MB-231 cells were treated for 24h with PTX (10nM) and OICR-9429 (20 μM) or Galunisertib (10 μM) and cell cycle was analyzed by flow cytometry for Bromodeoxyhuridine (BrDU) and PI content. Percentage (%) of cell population in each phase of the cell cycle (G0/G1, S and S-not cycling, G2/M) is reported within the panels.

Supplementary Figure S1

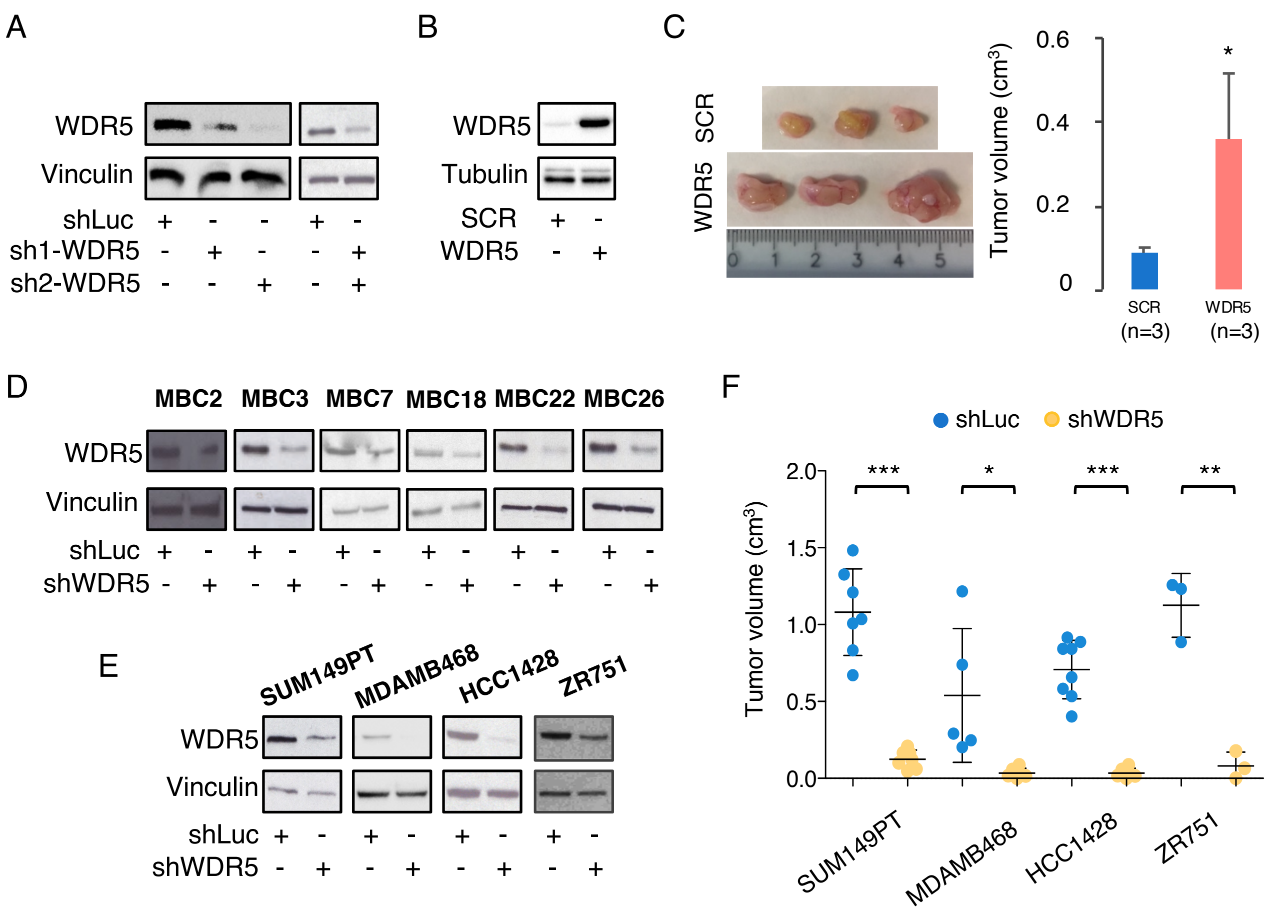

Supplementary Figure S2

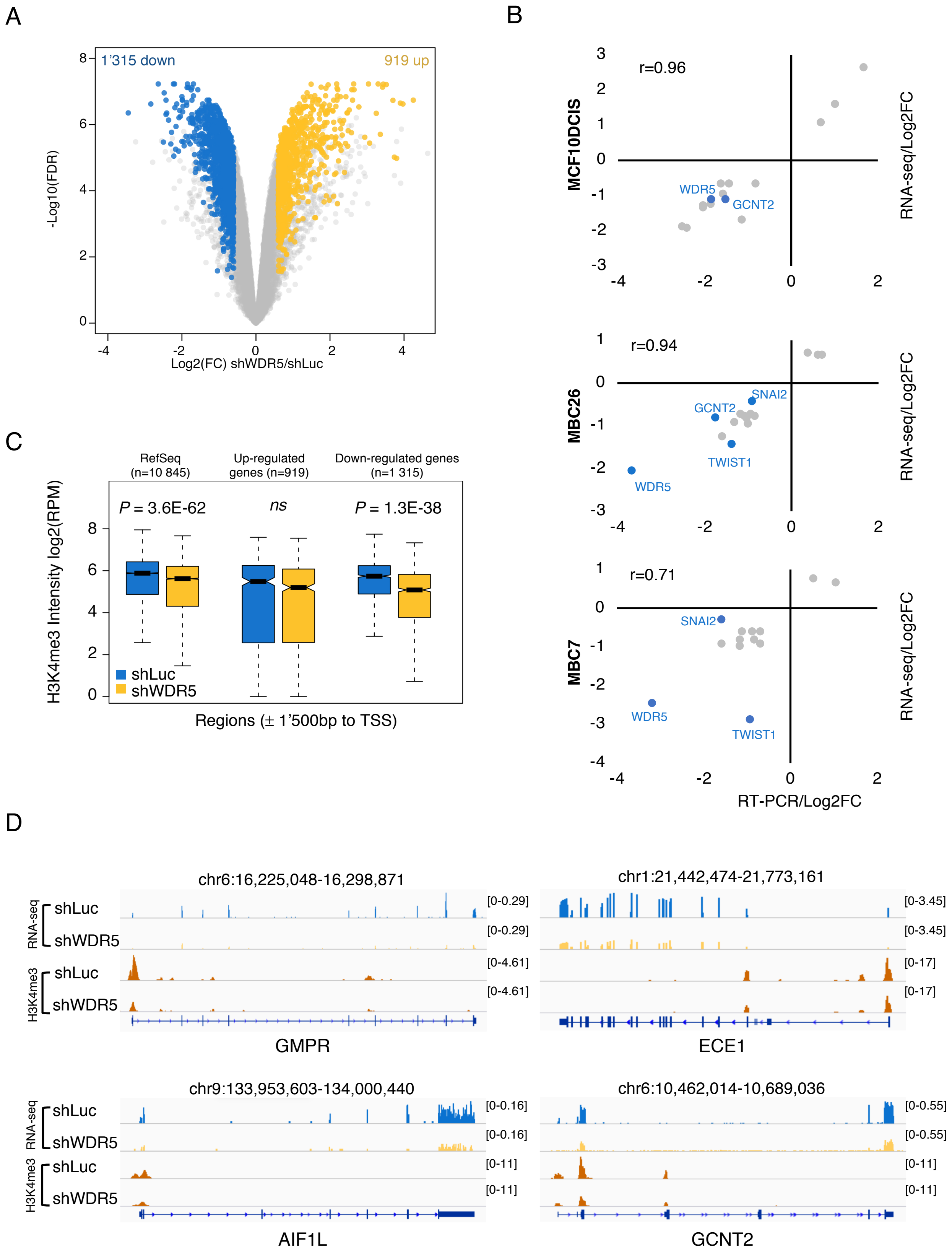

Supplementary Figure S3

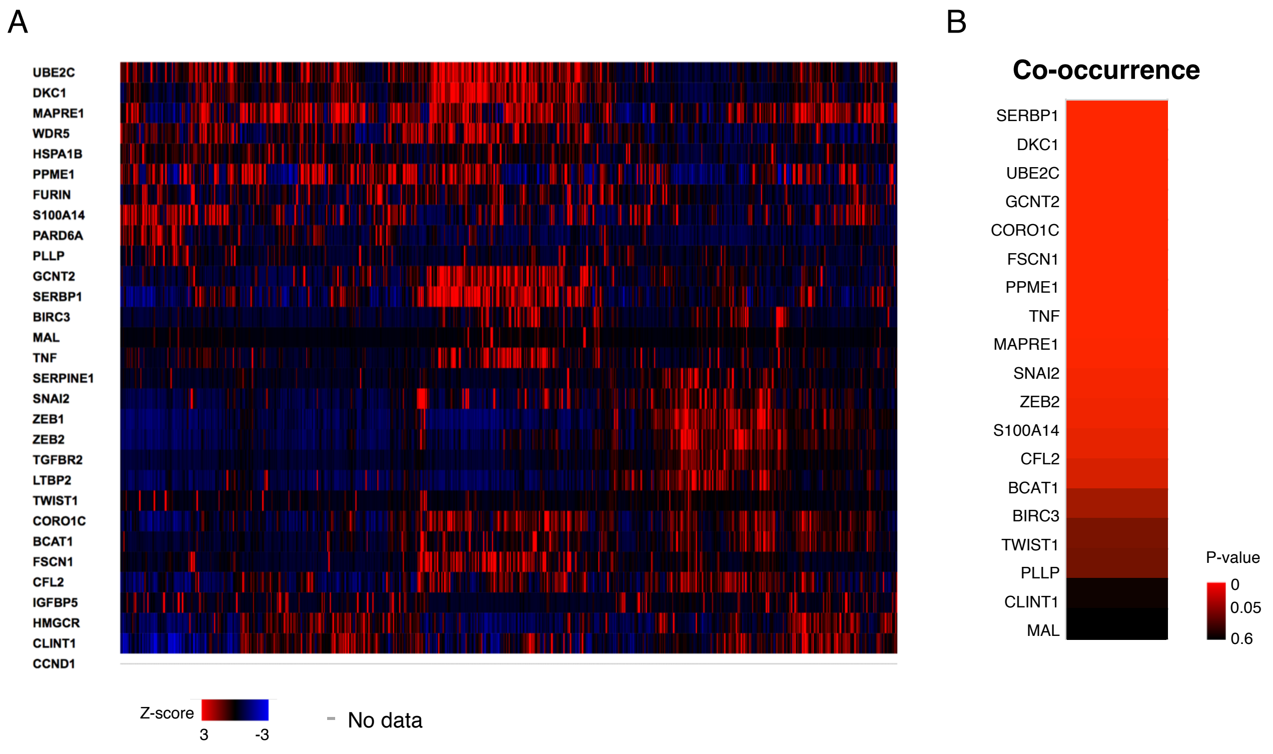

Supplementary Figure S4

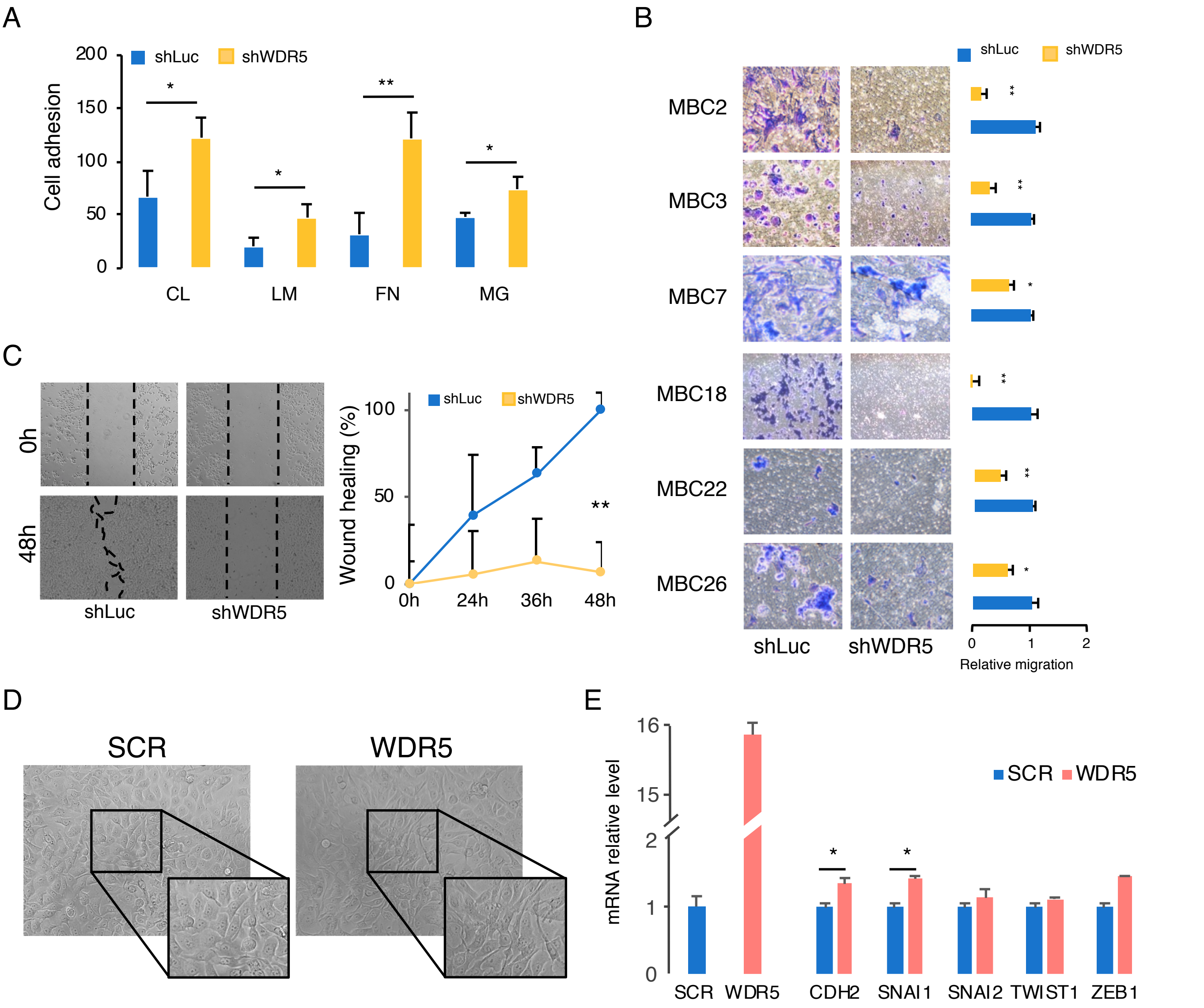

Supplementary Figure S5

A

Subgroup	HR	CI (95%)	*P* values
Main	1.35	1.16-1.58	1.50E-04
ER status	1.29	1.09-1.53	3.90E-03
Chemotherapy	1.36	1.16-1.58	1.00E-04
Endocrine therapy	1.34	1-15-1.57	2.00E-04

B

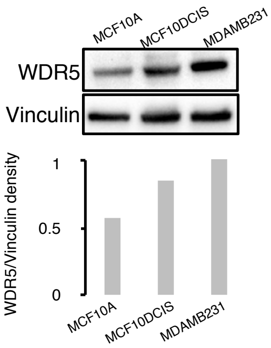

C

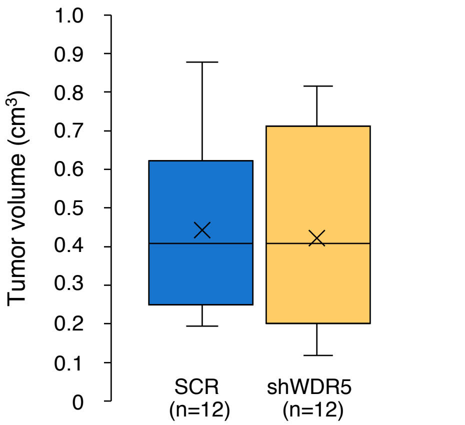

D

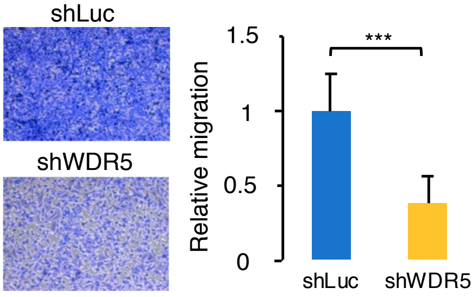

E

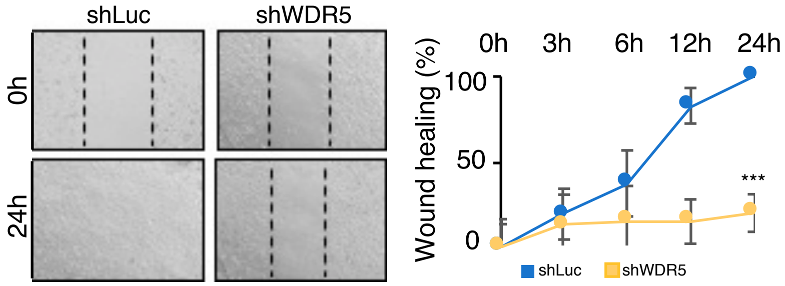

F

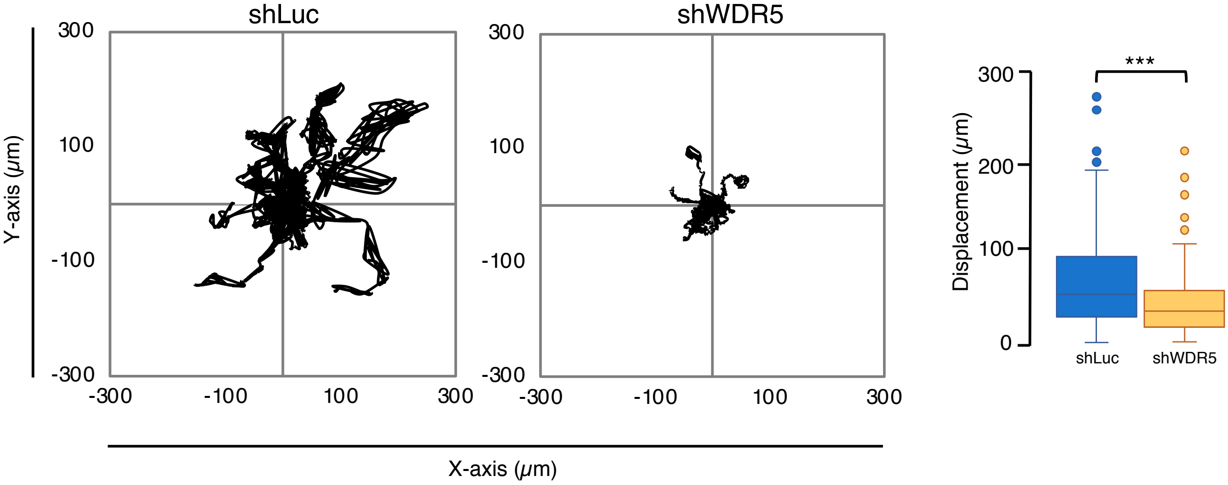

Supplementary Figure S6

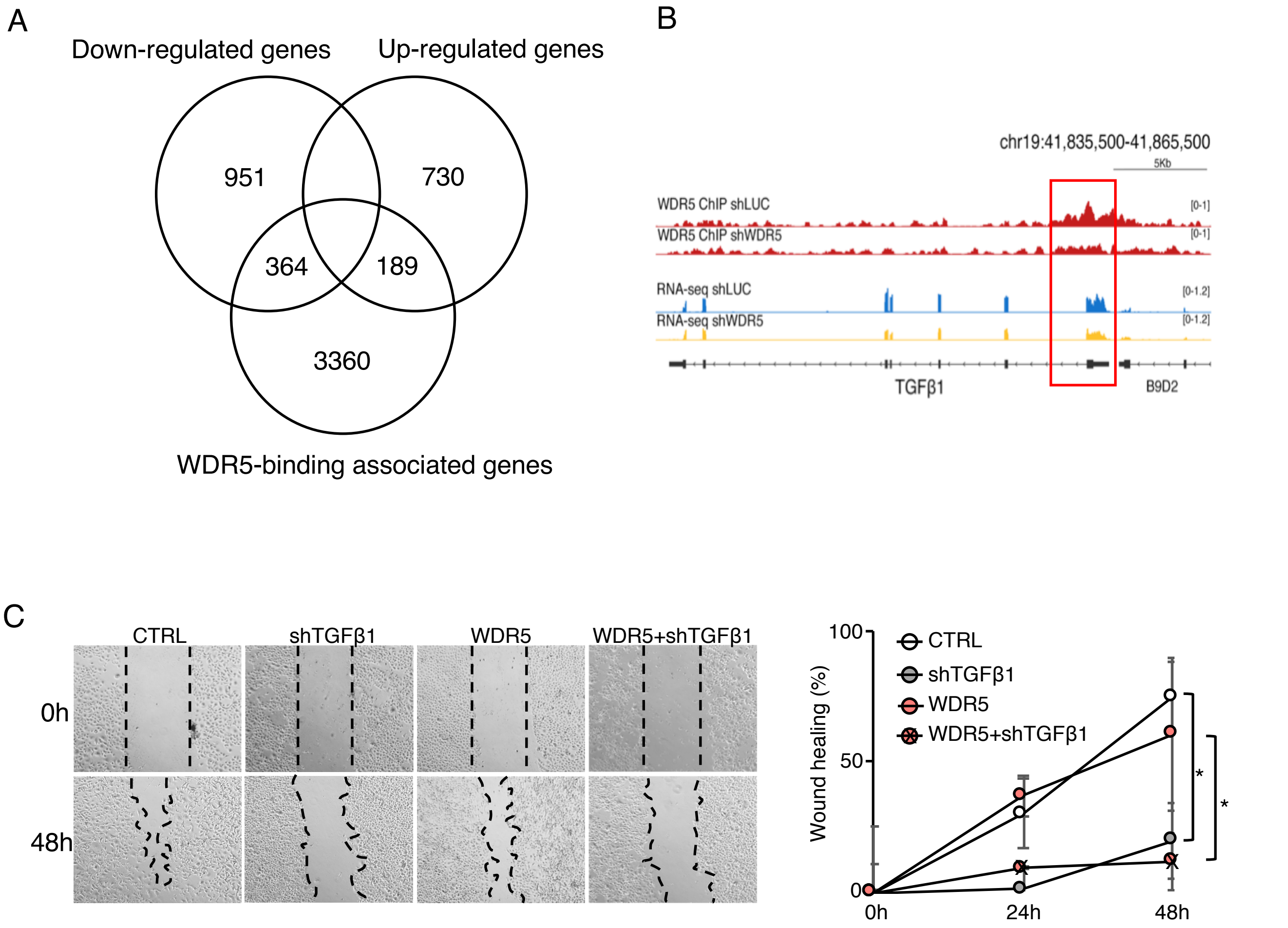

Supplementary Figure S7

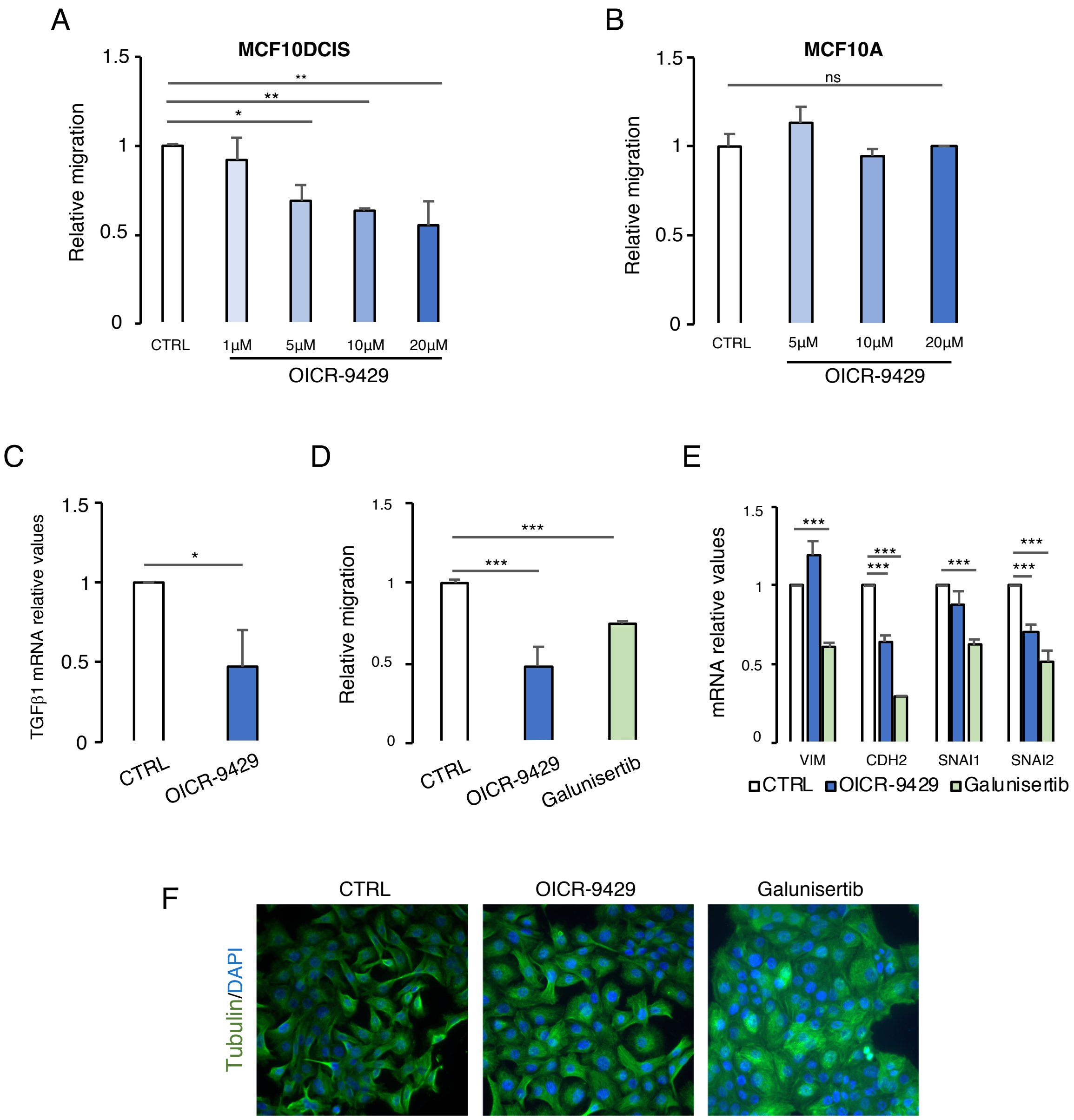

Supplementary Figure S8

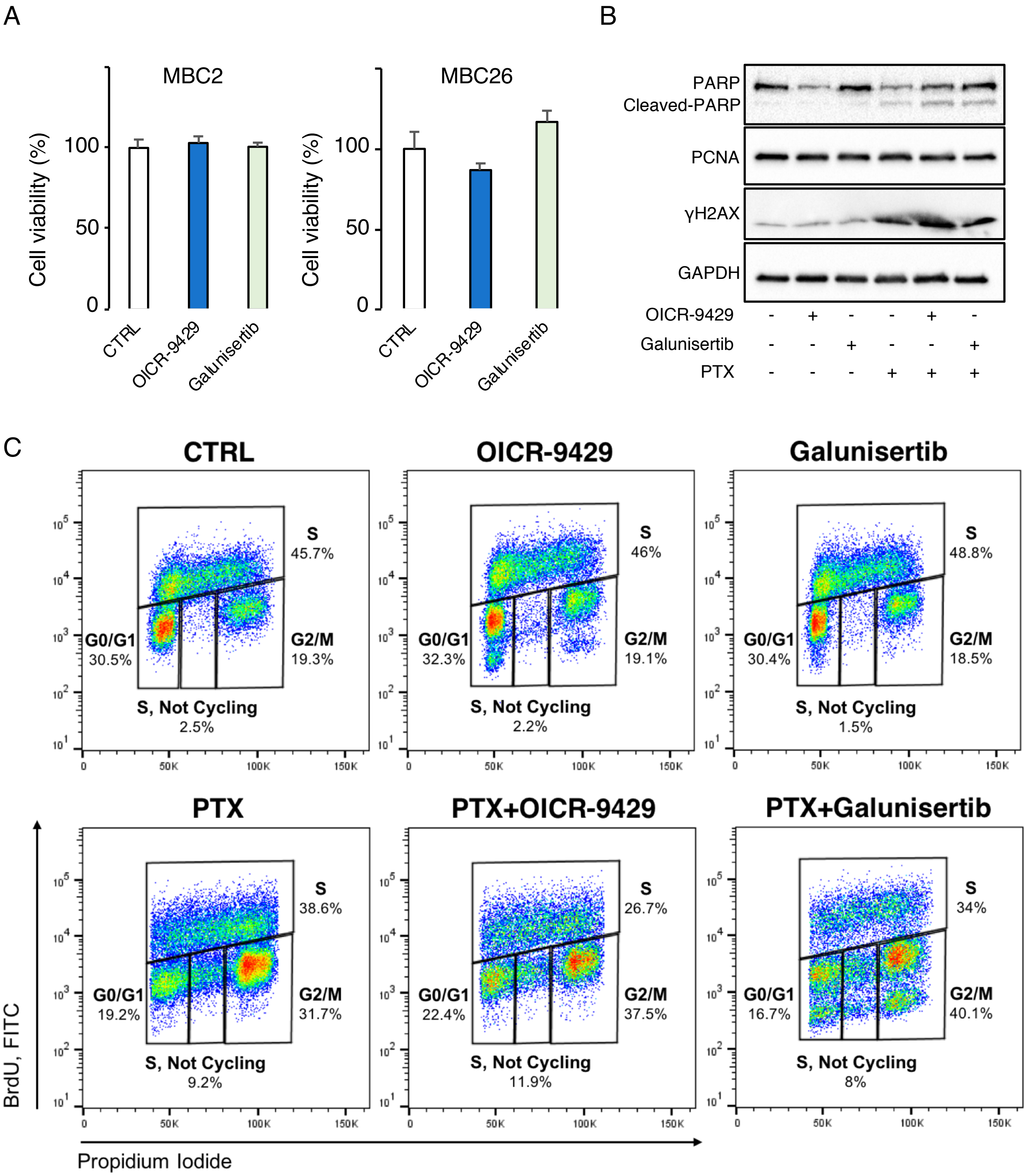

Supplement: Supplementary file 1 — Additional file 1: Figure S1. Effect of WDR5 silencing in Luminal B and Triple Negative breast cancer cell lines. Figure S2. WDR5 transcriptionally regulates its targets. Figure S3. WDR5-target genes are regulated in a larger cohort of patients. Figure S4. WDR5 regulates adhesion, migration and polarization of breast cancer cells. Figure S5. WDR5 silencing significantly inhibits migration of MDA-MB-231 breast cancer cells. Figure S6. TGFβ1 is transcriptionally regulated by WDR5. Figure S7. WDR5 inhibition reduces migration of breast cancer cells but not of non-transformed MCF10A breast cells. Figure S8. WDR5-TGFβ1 inhibition combined with Paclitaxel causes cell cycle block in MDA-MB-231 breast cancer cells. [file 13058_2019_1216_MOESM1_ESM.pdf]
