# Supplementary material for: WDR5 inhibition halts metastasis dissemination by repressing the mesenchymal phenotype of breast cancer cells
Source: Breast Cancer Res. 2019 Nov 21;21:123. doi: 10.1186/s13058-019-1216-y (PMC6873410; doi:10.1186/s13058-019-1216-y)
Supplement: Supplementary file 2 — Additional file 2. Table S1. Patients' tumor characterization for site of metastasis, clinical markers and treatments at the moment of relapse is reported. MBC: metastatic breast cancer. ER: Estrogen Receptor. PgR: Progesterone Receptor. LB: Luminal B. TN: Triple Negative. HER2 negative (-). ViFuP: Vinorelbine/ Fluorouracil/ Cisplatin. BEXE: Bevacizumab/ Capecitabine/ Cyclophosphamide/ Erlotinib. Table S2. Biological characterization for the main clinical markers of each patient (PT) and the corresponding PDX at passage in mice used for experimental purpose. MBC: metastatic breast cancer. LB: Luminal B. TN: Triple Negative. Table S3. List of 253 genes from MBC PDXs obtained by in common differentially expressed genes in at least two comparisons is reported. Gene-Ontology analysis by using DAVID tool (version 6.8 Beta) and Ingenuity Pathway Analysis (IPA) for upstream regulators were performed on PDXs. Differentially expressed genes down- and up-regulated in MCF10DCIS breast cancer cell line are shown. IPA for upstream regulators was also performed on MCF10DCIS. H3K4me3 ChIP-seq was performed on MCF10DCIS breast cancer cell line. Reads were mapped to the promoter region (±1500 bp relative to TSS) for annotated transcripts. Significant differential H3K4me3 values are shown. WDR5 genome wide binding sites on MCF10DCIS were assigned to the nearest proximal and distal transcription start sites (TSS)( ± 3kb). Table S4. RT-PCR primer sequences 5'--->3' are reported. [file 13058_2019_1216_MOESM2_ESM.pdf]

Supplementary Table S1.

Patients	Site of metastasis	Diagnosis	Characterization				Subtype	Treatment at relapse
			ER	PgR	HER2	Ki67		
**MBC2**	lung	2013	0%	0%	-	50%	TN	ViFuP
**MBC3**	lung	2013	>95%	0%	-	32%	LB	FULVESTRANT
**MBC7**	liver	2012	1%	<1%	-	83%	TN	BEXE
**MBC10**	lung	2014	90%	0%	-	40%	LB	LETROZOLE
**MBC18**	liver	2012	95%	0%	-	80%	LB	LETROZOLE
**MBC22**	liver	2012	95%	95%	-	25%	LB	CAPECITABINE
**MBC26**	liver	2012	75%	1%	-	59%	LB	TAXOL

Supplementary Table S2.

		ER	PgR	HER2	Ki67	SUBTYPE
**MBC2**	**PT**	0%	0%	0%	50%	TN
	**PDX4**	0%	0%	0%	85%	TN
**MBC3**	**PT**	>95%	0%	1 (50%)	32%	LB
	**PDX3**	>95%	5%	2 (40%)	45%	LB
**MBC7**	**PT**	1%	<1%	0%	83%	TN
	**PDX4**	1%	1%	0%	75%	TN
**MBC10**	**PT**	90%	0%	1(20%)	40%	LB
	**PDX2**	>95%	0%	1(10%)	65%	LB
**MBC18**	**PT**	95%	0%	1 (10%)	80%	LB
	**PDX2**	95%	10%	1(10%)	85%	LB
**MBC22**	**PT**	95%	95%	0%	25%	LB
	**PDX3**	95%	90%	0%	38%	LB
**MBC26**	**PT**	75%	1%	1 (40%)	59%	LB
	**PDX3**	>95%	0%	2(50%)	70%	LB

Supplementary Table S3. (See Excel file Table S3)

Supplementary Table S4.

**ECE1**	
FORWARD	CTCACCCAAGGCCTTAAACT
REVERSE	GTTCCCGTCCTTGTCATACTC
**HSPA1B**	
FORWARD	CTACCATTGAGGAGGTGGATTAG
REVERSE	CAAAGAAGTGAAGCAGCAAAGA
**PNP**	
FORWARD	GCAATAATCTGTGGTTCTGGATTAG
REVERSE	CATGACCTGGCACTGTACTT
**CEMIP**	
FORWARD	TCCTGCACCTTGGATTTAGAC
REVERSE	GAGCCTCGATGTCCATGATATT
**AIF1L**	
FORWARD	AAGGCGAGATTGACCTGATG
REVERSE	TCCTGTCACCTCTGAGATCAT
**GBP1**	
FORWARD	GGCTATGGACCAACTGTACTATG
REVERSE	AGCTGAATCCTCAACCTCATTC
**HMOX1**	
FORWARD	TGCTCAACATCCAGCTCTTT
REVERSE	TGCCACATTAGGGTGTCTTC
**IL32**	

FORWARD	GTCTCTCTCGGCTGAGTATTTG
REVERSE	CTCTTTGAAGTCGTCCTCCAG
**TMEM184B**	
FORWARD	CCCATTGAGTCCAGCTGTATG
REVERSE	GCTGACGGAGATGTTGTAGATG
**TNFAIP3**	
FORWARD	TCATCCACAAAGCCCTCATC
REVERSE	CATCATTCCAGTTCCGAGTATCA
**CALB2**	
FORWARD	CGCAGACGGAAATGGGTATATT
REVERSE	ACATCATGCCAGAGCCTTTC
**GCNT2**	
FORWARD	ACTCAATAGGATTCCAGGTGTTC
REVERSE	GTCTTCCATGTCACTCCACTTTA
**GMPR**	
FORWARD	TGTCACAGCACTCCATGTTTA
REVERSE	TGAACTCACGGCTACATTCTG
**DKC1**	
FORWARD	AACCACAAGGATGAGAGTTACC
REVERSE	GGCATTTACTGCACTGTCTTTC
**WDR5**	
FORWARD	CACAAGCTGGGAATATCCGATG
REVERSE	GGGGATTGAAGTTGCAGCAAAA
**VIM**	
FORWARD	AAAGTGTGGCTGCCAAGAAC

REVERSE	AGCCTCAGAGAGGTCAGCAA
**CDH2**	
FORWARD	GGGATCAAAGCCTGGAACATA
REVERSE	GACACGATTCTGTACCTCAACA
**SNAI2**	
FORWARD	CTTCCTGGTCAAGAAGCATTTC
REVERSE	GGGAAATAATCACTGTATGTGTGT
**SNAI1**	
FORWARD	ACTATGCCGCGCTCTTTC
REVERSE	GCTGGAAGGTAAACTCTGGATTA
**TWIST1**	
FORWARD	ACTGGACACACATACAGTGATTAT
REVERSE	GAATGGAGCAGCGGTAGTC
**TGFβ1**	
FORWARD	CGTGGAGCTGTACCAGAAATAC
REVERSE	CACAACTCCGGTGACATCAA
