# Supplementary material for: WDR5 inhibition halts metastasis dissemination by repressing the mesenchymal phenotype of breast cancer cells
Source: Breast Cancer Res. 2019 Nov 21;21:123. doi: 10.1186/s13058-019-1216-y (PMC6873410; doi:10.1186/s13058-019-1216-y)
Supplement: Supplementary file 3 — Additional file 3. Supplementary methods. [file 13058_2019_1216_MOESM3_ESM.pdf]

Supplementary methods

Plasmid and infection. shRNAs were cloned into the pRSI-U6-(sh)-UbiC-TagRFP-2A-Puro vector (Cellecta Inc.) and used to infect cells to silence a neutral control Luc (5'-CAAATCACAGAATCGTTGTAT-3') or WDR5 (sh#1: 5'-CTGGTTACAAGTTGGGAATAT-3'; sh#2: 5'-GTGTCTGGCTTAGAGGATAAT-3'). Concentrated lentiviral particles from single plasmids were produced by transfecting 293T cells and added as shRNAs pool to cell lines and PDXs cultures, together with 4 µg/mL polybrene (Sigma) for 16 hours. After 48 hours medium was replaced and 3 µg/mL of puromycin was added for 72 hours before performing the experiments. Cells were infected at high Multiplicity Of Infection (MOI) (Cell lines at MOI of ~3, and PDX culture cells at MOI of ~50). Over-expression vectors pHAGE-GFP_IRES_GFP or pHAGE-WDR5_IRES_GFP was obtained from MD Anderson Cancer Center (Texas). ShRNA sequences to silence TGFβ1 were extrapolated elsewhere [1] and cloned into pRSI-U6-(sh)-UbiC-TagGFP-2A-Puro vector (Cellecta Inc.). MCF10DCIS cells were transduced as indicated before.

***In vitro* study.** Proliferation assay. 2000 MCF10DCIS cells *per* well were seeded in triplicate. Cell Titer Glo (Promega) was used to evaluate cells viability from day 0 to day 3 after plating. Data are expressed as relative ratio of treated (shWDR5) and control cells (shLuc). FBS-directed migration assay. ShLuc and shWDR5 cells were seeded ($2.5x10^5$ MCF10DCIS or PDXs cells in duplicate; $5x10^4$ MDA-MB-231 and MCF10A) in the upper chamber of 8.0 µm pore size inserts of 24-well plates. MCF10DCIS and MCF10A cells were resuspended in 0.5% horse serum, PDXs in 1% FBS, and complete medium supplemented with 50% FBS were added as chemoattractant in the lower chamber, instead MDA-MB-231 were starved overnight in 1% FBS and complete medium was added in the lower chamber. After 16 (MCF10DCIS and MDA-MB231) to 36 hours (PDXs)

of incubation, migrated cells were fixed and stained with 0.5% Crystal Violet. Migration was quantified by ImageJ analysis. Wound healing assay. MDA-MB-231 and MCF10DCIS cells were seeded on six-well plates and a scratch was introduced into a confluent monolayer with a pipette tip. To ensure that differences in cell migration were not due to differences in cell growth, cells were incubated overnight in serum-free medium prior to scratch injury. Images were taken immediately or at regular intervals until 24h (MDA-MB-231) or 48h (MCF10DCIS) from scratching. Cell migration rate was quantified by ImageJ as distance between migration front and closure of the wound. Live cell random migration assay. 1.2 x 10^4 MCF10DCIS or MDA-MB-231 cells were plated in 12 well plates and time-lapse imaging of cell migration was performed on a Nikon Eclipse Ti microscope equipped with an incubator chamber maintained at 37°C in a 5% CO_2 atmosphere. Tracking of cells was performed using the Trackmate plugin distributed by Fiji software. Four random fields were acquired *per* well and about 200 cells were measured *per* group (acquisition every 10' for 24h). The motility of cells for the two groups (shLuc and shWDR5) was considered by excluding cells' trajectory due to mitotic events.

Western blot. Membranes were probed with the following antibodies: Vinculin (Sigma V9131), GAPDH (Cell Signaling #2118), Tubulin (Sigma Aldrich T9026), WDR5 (Cell Signaling #13105), Vimentin (Abcam), CDH1 [24E10] (Cell signaling), CDH2 [5D5] (Abcam), SNAI2 [C19G7] and SNAI1 [C15D3] (both from Cell Signaling), PCNA [M0879] (Dako), PARP (Cell Signaling) and γH2AX (Biolegend).

Subcellular fractionation. Subcellular fractionation in shLuc and shWDR5 MCF10DCIS cells was performed as indicated by supplier (Subcellular fractionation kit - Thermo Scientific).

Immunohistochemistry. Eleven normal mammary glands from patients and tumor fragments from eight PDXs were formalin-fixed and paraffin-embedded. After deparaffinization, sections were treated with citrate for 50 minutes at 95°C, followed by incubation with 3% hydrogen peroxide in distilled water for 5 minutes at RT. Sections were stained with WDR5 primary antibody (Cell Signaling #13105). Images were acquired by Olympus BX51 up-right (objective 40x) connected to Nikon Color Camera (software NIS-elements). Expression was quantified by using Fiji tools for DAB positivity. Statistical significance was determined using a Student *t* test.

Cell cycle analysis. MDA-MB-231 cells were treated with PTX (10 nM), OICR-9429 (20 µM) or Galunisertib (10 µM) alone or in combination for 24 hours. Cells were pulsed with 5 mM Bromodeoxyuridine (BrdU), fixed and stained against BrdU (BD Biosciences). Cells were stained with secondary antibody, incubated with propidium iodide (PI) and RNaseA and then acquired by fluorescent-activated cell sorting (FACS) at FACSCelesta (BD Bioscience). Analysis was performed using FlowJo 10.6 analysis software.

RNA-sequencing. Libraries were sequenced in multiplex at 50bp single reads on an Illumina HiSeq2000 (average sequence coverage ~40 million reads/sample). For PDXs, after quality filtering according to the Illumina pipeline, 50 bp single-end reads were aligned to the human (hg19) and mouse (mm10) reference genome using TopHat2 (version 2.1.0) [2] with the option "*--b2-very-sensitive*". After removing the reads aligned to the mouse genome, only uniquely mapped reads were retained. Alignment to the hg19 human genome was performed on MCF10DCIS cells. At gene level, expression counts were estimated using featureCounts (Rsubread version 1.5.1) [3], summarized across all

exons as annotated in the *Homo sapiens* transcriptome (NCBI build 37.2), with default options. Both coding and long noncoding RNA genes were retained for downstream analyses. Normalization and identification of differentially expressed genes in three biological replicates of shLuc control and in shWDR5 for each PDX or in the MCF10DCIS cancer cell line, were carried out using EdgeR R-package (version 3.2.2) [4]. Prior to normalization using the Trimmed Mean of M (TMM) method, genes whose expression was lower than 0.1 Count Per Million (0.5 for the MCF10DCIS) in more than three samples were filtered out. A common dispersion was estimated for all genes to measure the global biological variation (with option *robust = "TRUE"*). A negative binomial generalized log-linear model was fitted to each gene, and likelihood ratio tests were performed to assess differential expression in pairwise analyses for each PDX [5]. The expression levels were calculated using the reads per kilobase per million reads method (RPKM). Genes were identified as differentially expressed (DEGs) when the following criteria were met: $\log_2$ fold-change (FC) $\geq |0.6|$, false discovery rate (FDR) < 0.05 and expression > 0.5 RPKM (1 RPKM in the MCF10DCIS cell line) in all sample in at least one condition. 253 genes (161 down- and 92 up-regulated) were commonly deregulated in at least 2 comparisons. This set was used in the functional category enrichment analyses.

DEGs in PDX samples were hierarchically clustered using pheatmap R package [Kolde R: pheatmap: Pretty Heatmaps 2015. https://CRAN.R-project.org/package=pheatmap] utilizing a Euclidean distance metric and complete linkage rule, after removing batch effects across PDX, setting the minimum RPKM value to 0.1 and log2-transformation. DEGs were analyzed using Ingenuity Pathway Analysis (IPA, Ingenuity® Systems, www.ingenuity.com; Redwood City, CA) and Gene Ontology term enrichment using DAVID tool (version 6.8 Beta) [6].

ChIP-sequencing. Reads were mapped to the human hg19 reference genome using Bowtie2 v2.2.6 [7] with the "*–very-sensitive*" preset of parameters. Reads that did not align to the nuclear genome or that aligned to the mitochondrial genome were removed. Moreover, duplicate reads were marked and removed using SAMtools [8]. Peak calling for WDR5 ChIP-seq was performed using MACS2 (version 2.1.0.20150731) [9] using the "--*nomodel*", *"--extsize 200*" and "*--pvalue 0.001*" flags and arguments. Peaks with a *P*-value > 1E-3, both in the comparison ChIP vs. input DNA and ChIP vs. ChIP, and those blacklisted by the ENCODE consortium analysis of artefactual signals in human genome (https://sites.google.com/site/anshulkundaje/projects/blacklists) were removed using bedtools [10]. The annotatePeaks script from the HOMER package [11] was used to identify the genomic location of WDR5 peaks and assign them to the nearest transcription start site (TSS). Then we classified each peak as either TSS-proximal or TSS-distal, depending on its distance (< or > +/-3 kb, respectively) from TSS.

The H3K4me3 ChIP-seq of shLuc control and shWDR5 in MCF10DCIS cancer cell line were aligned as above. Then, we extracted reads mapped to a regions of ±1'500 bp relative to TSS for all annotated transcripts (NCBI build 37.2) using the coverageBed tool in bedtools [10].

We applied RPM normalization to all datasets and tracks for visualization in the Integrative Genomics Viewer (IGV) [12] were generated using bedGraphToBigWig tool.

ChIP Quantitative PCR. Real-time PCR and primers specific to the promoter of TGFβ1 (5'-3'. FW: CTTCCTCCAGCCAGTTTCTT; RV: TCACCCGCGTGCTAATG) were used to determine TGFβ1 binding to the immunoprecipitated DNA with WDR5 antibody. AchR (acetylcholine receptor) was used as negative control. The intensity was normalized with respect to the Input (no immunoprecipitation).

References

1. Oh S, Kim E, Kang D, Kim M, Kim JH, Song JJ. Transforming growth factor-beta gene silencing using adenovirus expressing TGF-beta1 or TGF-beta2 shRNA. Cancer gene therapy. 2013;**20**(2):94-100.

2. Kim D, Pertea G, Trapnell C, Pimentel H, Kelley R, Salzberg SL. TopHat2: accurate alignment of transcriptomes in the presence of insertions, deletions and gene fusions. Genome biology. 2013;**14**(4):R36.

3. Liao Y, Smyth GK, Shi W. featureCounts: an efficient general purpose program for assigning sequence reads to genomic features. Bioinformatics (Oxford, England). 2014;**30**(7):923-30.

4. Robinson MD, McCarthy DJ, Smyth GK. edgeR: a Bioconductor package for differential expression analysis of digital gene expression data. Bioinformatics (Oxford, England). 2010;**26**(1):139-40.

5. Auton A, Brooks LD, Durbin RM, Garrison EP, Kang HM, Korbel JO, et al. A global reference for human genetic variation. Nature. 2015;**526**(7571):68-74.

6. Huang DW, Sherman BT, Tan Q, Collins JR, Alvord WG, Roayaei J, et al. The DAVID Gene Functional Classification Tool: a novel biological module-centric algorithm to functionally analyze large gene lists. Genome biology. 2007;**8**(9):R183.

7. Langmead B, Salzberg SL. Fast gapped-read alignment with Bowtie 2. Nature methods. 2012;**9**(4):357-9.

8. Li H, Handsaker B, Wysoker A, Fennell T, Ruan J, Homer N, et al. The Sequence Alignment/Map format and SAMtools. Bioinformatics (Oxford, England). 2009;**25**(16):2078-9.

9. Zhang Y, Liu T, Meyer CA, Eeckhoute J, Johnson DS, Bernstein BE, et al. Model-based analysis of ChIP-Seq (MACS). Genome biology. 2008;**9**(9):R137.

10. Quinlan AR, Hall IM. BEDTools: a flexible suite of utilities for comparing genomic features. Bioinformatics (Oxford, England). 2010;**26**(6):841-2.

11. Heinz S, Benner C, Spann N, Bertolino E, Lin YC, Laslo P, et al. Simple combinations of lineage-determining transcription factors prime cis-regulatory elements required for macrophage and B cell identities. Molecular cell. 2010;**38**(4):576-89.

12. Robinson JT, Thorvaldsdottir H, Winckler W, Guttman M, Lander ES, Getz G, et al. Integrative genomics viewer. Nature biotechnology. 2011;**29**(1):24-6.
